# Radiation-promoted CDC6 protein stability contributes to radioresistance by regulating senescence and epithelial to mesenchymal transition

**DOI:** 10.1038/s41388-018-0460-4

**Published:** 2018-08-29

**Authors:** Xiaohui Yu, Youhong Liu, Linglong Yin, Yongbo Peng, Yuchong Peng, Yingxue Gao, Bowen Yuan, Qianling Zhu, Tuoyu Cao, Bowen Xie, Lunquan Sun, Yan Chen, Zhicheng Gong, Yuanzheng Qiu, Xuegong Fan, Xiong Li

**Affiliations:** 1 Center for Molecular Medicine, Xiangya Hospital, Central South University, Changsha, China; 2 Hunan Key Laboratory of Molecular Radiation Oncology, Xiangya Hospital, Central South University, Changsha, China; 3 State Key Laboratory of Chemo/Biosensing and Chemometrics, Hunan University, Changsha, China; 4 Department of Pathology, The Third Xiangya Hospital, Central South University, Changsha, China; 5 Department of Pharmacy, Xiangya Hospital, Central South University, Changsha, China; 6 Department of Otorhinolaryngology, Xiangya Hospital, Central South University, Changsha, China; 7 Hunan Key Laboratory of Viral Hepatitis, Xiangya Hospital, Central South University, Changsha, China

**Keywords:** Predictive markers, Radiotherapy

## Abstract

Ionizing radiation (IR) is a conventional cancer therapeutic, to which cancer cells develop radioresistance with exposure. The residual cancer cells after radiation treatment also have increased metastatic potential. The mechanisms by which cancer cells develop radioresistance and gain metastatic potential are still unknown. In this study acute IR exposure induced cancer cell senescence and apoptosis, but after long-term IR exposure, cancer cells exhibited radioresistance. The proliferation of radioresistant cells was retarded, and most cells were arrested in G0/G1 phase. The radioresistant cells simultaneously showed resistance to further IR-induced apoptosis, premature senescence, and epithelial to mesenchymal transformation (EMT). Acute IR exposure steadily elevated CDC6 protein levels due to the attenuation of ubiquitination, while CDC6 overexpression was observed in the radioresistant cells because the insufficiency of CDC6 phosphorylation blocked protein translocation from nucleus to cytoplasm, resulting in subcellular protein accumulation when the cells were arrested in G0/G1 phase. CDC6 ectopic overexpression in CNE2 cells resulted in apoptosis resistance, G0/G1 cell cycle arrest, premature senescence, and EMT, similar to the characteristics of radioresistant CNE2-R cells. Targeting CDC6 with siRNA promoted IR-induced senescence, sensitized cancer cells to IR-induced apoptosis, and reversed EMT. Furthermore, CDC6 depletion synergistically repressed the growth of CNE2-R xenografts when combined with IR. The study describes for the first time cell models for IR-induced senescence, apoptosis resistance, and EMT, three major mechanisms by which radioresistance develops. CDC6 is a novel radioresistance switch regulating senescence, apoptosis, and EMT. These studies suggest that CDC6^high^KI67^low^ represents a new diagnostic marker of radiosensitivity, and CDC6 represents a new therapeutic target for cancer radiosensitization.

## Introduction

Nasopharyngeal carcinoma (NPC) is a common head and neck malignancy in Southern China, Southeast Asia, and Africa, where its incidence is higher than in western countries [1]. Ionizing radiation (IR) is a primary therapeutic approach for early NPC, which is usually highly radiosensitive, achieving a 5-year overall survival of 90 and 84% for stage I and IIA respectively [2]. However, cancer cells in some patients with advanced disease develop radioresistance and increased metastatic potential, resulting in the treatment failure and tumor relapse [3]. Currently, there are few effective biomarkers available in the clinic for predicting tumor radiosensitivity [4].

IR directly induced DNA lesions and chromosome breaks, resulting in cell death [5]. Some cells, however, developed radioresistance and escaped cell death. Mechanisms that may separately or synergistically contribute to radioresistance have been proposed [4]. Recent studies indicated that stress-induced premature senescence and epithelial−mesenchymal transition (EMT) contribute to radioresistance [6, 7]. In malignant epithelial cells, IR promoted invasion and metastasis by inducing EMT to bypass senescence [8, 9].

Cell division cycle 6 (CDC6) is an essential regulator of DNA replication. CDC6 overexpression has been detected in a number of cancer types, and high levels of CDC6 correlate with poor prognosis in cancer patients [10, 11] and radioresistance in cancer cells [12]. The ectopic overexpression of CDC6 leads to DNA hyper-replication, DNA damage, and genomic instability, which results in cell senescence [10, 13]. CDC6 overexpression promoted EMT by epigenetically suppressing E-cadherin expression [14]. Conversely, CDC6 knockdown promoted cell apoptosis [15]. Though CDC6 has been reported to be involved in tumor transformation [11, 16], cell apoptosis [17], senescence, and EMT, the full role of CDC6 in radiosensitivity remains to be determined.

In the present project, we observed cell senescence and apoptosis when cancer cells were exposed to IR, and we then established radioresistant cancer cells by long-term exposure to IR. Such characters as cell senescence and EMT were identified in the radioresistant cancer cells. The protein levels of CDC6 and Ki67, a cell proliferation marker were assessed in the radioresistant cancer cells as well as NPC tumor specimens. After the identification of CDC6 roles in radioresistance, we elevated CDC6 level in the parental cancer cells, or CDC6 was depleted in the radioresistant cancer cells, and observed the switch of cell senescence, EMT, and radiosensitivity. The IR sensitivity of tumor xenografts was tested when CDC6 was depleted in the radioresistant cancer cells. The present study is to identify the novel roles of CDC6 in cancer cell radiosensitivity.

## Results

### IR-induced cancer cell apoptosis and senescence

**Fig. 1:**
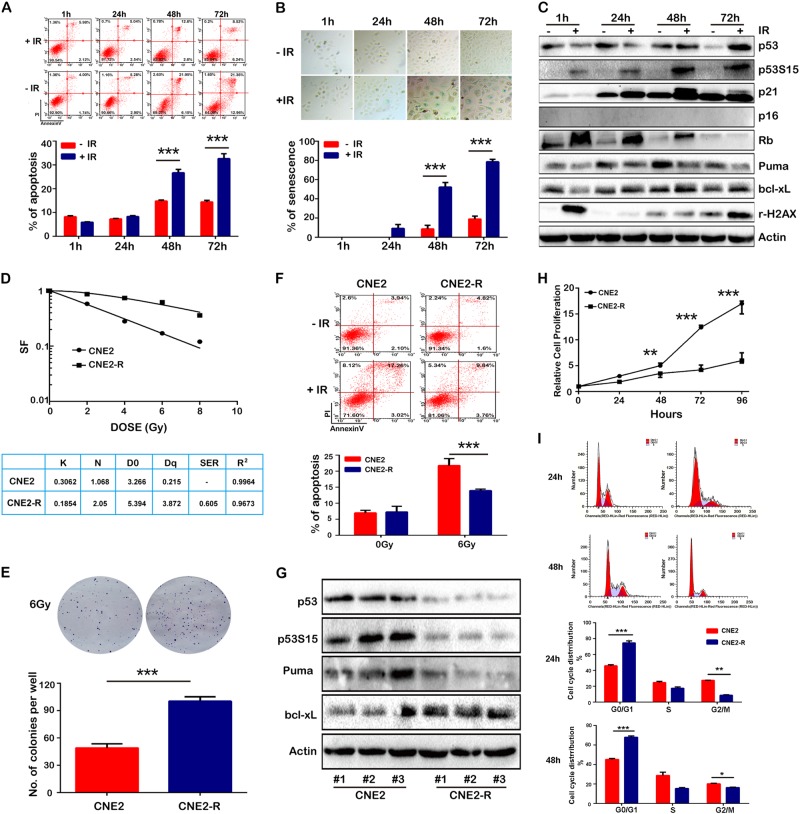
Acute IR exposure induced cell apoptosis and senescence, and cancer cells developed radioresistance after long-term IR exposure. CNE2 cells were exposed to 10 Gy X-ray radiation, and cell apoptosis and senescence were assessed 1, 24, 48, and 72 h after IR exposure. **a** Cells were stained with Annexin V-FITC/PI, and apoptotic cells were analyzed by flow cytometry. **b** Cell senescence was assessed by a senescence-associated β-galactosidase assay. **c** The protein levels of DNA damage, apoptosis, and senescence-associated molecules assessed by western blot. **d** CNE2 cells exhibited radioresistance after IR exposure with gradually increasing doses. Equal numbers of CNE2 and CNE2-R cells were exposed to 0, 2, 4, 6, and 8 Gy IR. The living cells formed cell colonies after 3 weeks. The radiobiological parameters D0, Dq, N, and sensitizing enhancement ratio (SER) were calculated and radiosensitivity was assessed by using a single-hit multitarget model of cell survival. **e** The cell colonies were stained by crystal violet after 6 Gy X-ray IR treatment. **f** CNE2-R cells showed resistance to IR-induced apoptosis. CNE2 and CNE2-R cells were exposed to 6 Gy X-ray IR, and cell apoptosis was detected by Annexin V-FITC/PI assay 24 h after radiation. **g** The protein levels of apoptotic molecules detected by western blot. Three repeats were used for each cell line. **h** The proliferation of CNE2-R cells was retarded compared to the parental CNE-2 cells. Cell viability was assessed by MTS assay, and the living cells were counted 24, 48, 72, and 96 h after cell seeding. **i** The cell cycle distribution of CNE2 and CNE2-R was analyzed 24 and 48 h after cell seeding by cell cycle detection kit. *P < 0.05, ** *P* < 0.01, *** *P* < 0.001

The manners of IR-induced cell death include programmed cell death apoptosis, and permanent cell growth arrest senescence [18]. IR (10 Gy) induced about 26.8% apoptotic CNE2 cells at 48 h and 33.3% at 72 h (Fig. 1a), vs. only 12% apoptotic U2OS cells at 48 h and 32% at 72 h after IR exposure (Supplementary Figure 1A). In addition to apoptosis, IR induced around 54% CNE2 cell senescence at 48 h, up to 79% at 72 h (Fig. 1b). Similar results were observed in U2OS cells. 6 Gy of IR induced 32% senescent cells at 48 h, up to 56% at 72 h (Supplementary Figure 1B). We continuously monitored the expression of apoptosis- and senescence-associated molecules when cancer cells were exposed to IR. As shown in Fig. 1c, IR quickly induced DNA damage (phosphorylated γ-H2AX) in CNE2 cells at 1 h, and DNA damage was repaired at 24 h. However, DNA damage intensified from 48 to 72 h, suggesting an unknown mechanism resulting in a second DNA damage. p53 and downstream molecule p21 protein levels were steadily elevated, p53 was activated (phosphorylation of p53 Ser 15) over the time of IR exposure. p21 is closely associated with cell cycle arrest and cell senescence [19]. The expression of the p53-dependent proapoptotic molecule PUMA decreased, while the antiapoptotic molecule Bcl-xL increased. Similar results were found in U2OS cells (Supplementary Figure 1C). Both CNE2 and U2OS cells are deficient in tumor suppressor gene *p16*, and IR exposure did not increase p16 expression to a visible level within 72 h after IR exposure. It is well known that p16, p53, and p21 are three major markers of cell senescence [20]. The results indicated that p53 and p21 probably were required for IR-induced senescence in p16-deficient cancer cells.

### Radioresistant cancer cells developed apoptosis resistance, inhibited cell proliferation, and were arrested in G0/G1 cell cycle phase

In previous studies, we generated a radioresistant cell line CNE2-R [21]. The radioresistance of CNE2R cells was validated (Fig. 1d). At the dose of 6 Gy IR, CNE2-R formed many more cell colonies than CNE2 cells (*P* < 0.001, Fig. 1e). IR (6 Gy) induced 23.3% apoptotic CNE2 cells, vs. only 14.6% apoptotic CNE2-R cells at 24 h after IR exposure (Fig. 1f). Unexpectedly, the protein levels of p53 and phosphorylated p53 did not rise, but significantly declined in CNE2-R cells compared to CNE2 cells. The proapoptotic molecule PUMA declined while antiapoptotic molecule Bcl-xL elevated, which might contribute to apoptosis resistance (Fig. 1g).

The proliferation of CNE2-R cells was retarded compared to CNE2 cells (Fig. 1h), and more CNE2-R cells were arrested in G0/G1 phase than CNE2 cells (46.4 vs. 74.2% at 24 h, and 44.5 vs. 69.1% at 48 h, Fig. 1i). It is well known that the cells in individual cell cycle phase respond to IR at different degrees. Cells in G2/M phase are more sensitive to IR than those in S phase, and G0/G1 phase cells are the most resistant to IR [22]. Therefore, long-term IR exposure arrested more cells in G0/G1 phase, which retarded cell proliferation and promoted apoptosis resistance.

### Radioresistant cancer cells simultaneously exhibited premature senescence and EMT phenotype

**Fig. 2:**
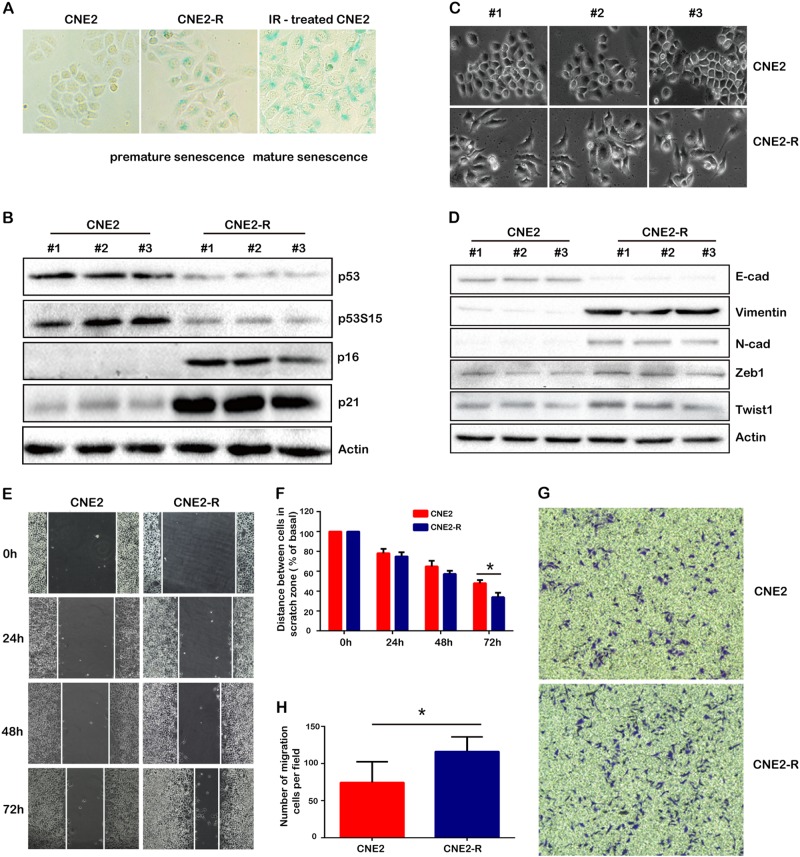
CNE2-R cells underwent premature cell senescence and EMT. **a** CNE2-R cells exhibited premature senescence. CNE2 or CNE2-R cells were stained with β-galactosidase and positive cells were compared (100×). **b** Senescence-associated proteins were assessed by western blot. **c** The morphology of CNE2 and CNE2-R cells was compared, and CNE2-R exhibited typical EMT phenotype. **d** EMT-associated proteins including E-cadherin, Vimentin, N-cadherin, twist, and Zeb1 were assessed by western blot. **e**, **f** Cell migration (scratch wound healing) assay. Confluent monolayer of cells was scratched, displaced cells were washed away and the cell gaps were measured at 24, 48, and 72 h after scratching. **g**, **h** Cell invasion (transwell) assay. Cells were starved for 48 h and re-plated on transwell plate inserts with serum-free media, with culture media with 10% FBS set on the bottom wells. The invasive cells on the membrane were stained with violet crystal 24 h after cell plating. *P < 0.05

CNE2-R cells exhibited the characteristics of senescence, such as apoptosis resistance, retarded cell proliferation, and G0/G1 cell cycle arrest. The SA-β-Gal assay was used to assess the degree of cell senescence. As shown in Fig. 2a, more β-Gal-positive cells were observed in CNE2-R cells than CNE2 cells, but the positive cells rarely exhibited typical and mature senescence morphology, such as flat and enlarged cell size. We randomly selected three cell colonies from CNE2 and CNE2-R respectively, and tested the expression of senescence-associated proteins, such as p16, p21, and p53. p16 expression was easily detected in CNE2-R cells, and undetectable in CNE-2 cells (Fig. 1c). In contrast, the expression level of p21 significantly increased, while p53 and phosphorylated p53 decreased (Fig. 2b). The inconsistency of p53, p16, and p21 expression indicated that CNE2-R cells were undergoing p53-independent premature senescence, which contributed to the formation of radioresistant CNE2-R cells.

The cell morphology of CNE2 and CNE2-R is much different. Compared to CNE2 cells, the levels of E-cadherin significantly declined in CNE2-R cells, while the levels of Vimentin, N-Cadherin, and the critical EMT transcription factors Twist and Zeb1 significantly rose (Fig. 2d). These data indicated that the radioresistant CNE2-R cells underwent EMT. We also observed EMT in another radioresistant NPC cell line HK1-R (Supplementary Figure 2A and B). As we expected, the cell migration and invasion capabilities of CNE2-R were significantly stronger compared to CNE2 cells by scratch wound healing assay (Fig. 2e, f) or transwell assay (Fig. 2g, h). It was reported that EMT would increase the subpopulation of cancer stem cells (CSC) [23]. Compared to CNE2 cells, the percentage of CSC (CD44^+^CD24^+^) significantly increased in CNE2-R cells (6.83 vs. 0.06%) (Supplementary Figure 2C).

### “Acute” or “chronic” IR exposure elevated CDC6 protein levels, and high CDC6 levels were detected in partially IR-responsive (radiation-resistant) NPC tumor tissues

**Fig. 3:**
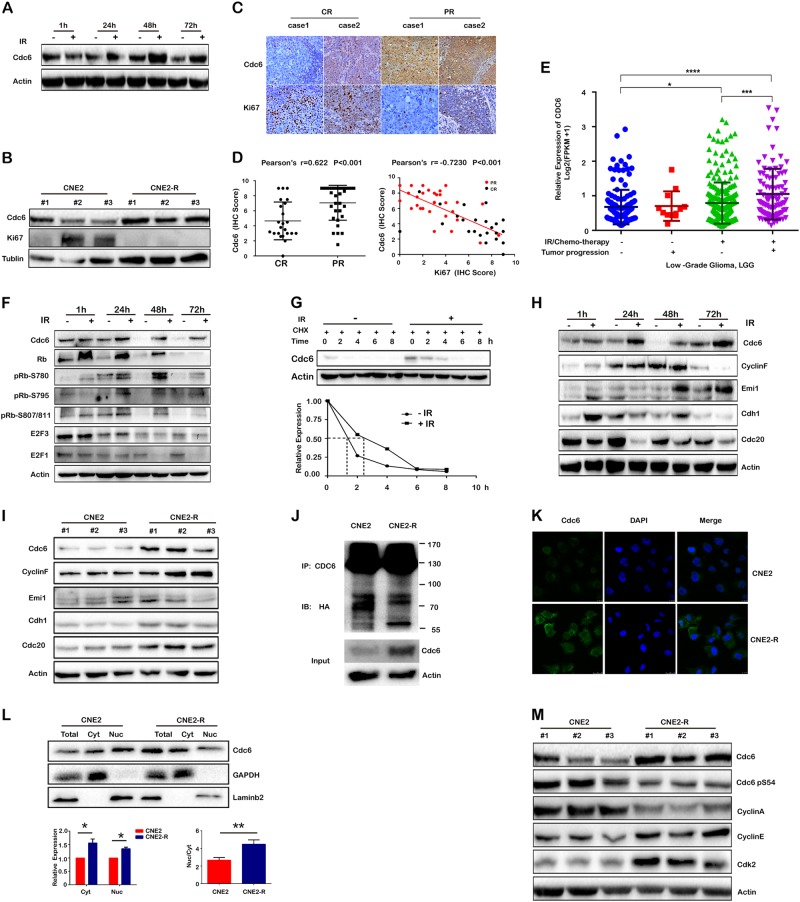
Acute IR exposure elevated CDC6 protein levels by ubiquitin-proteasome pathways, and chronic IR elevated CDC6 protein levels by decreasing CDC6 phosphorylation-induced nuclear-cytosolic translocation. **a** CNE2 cells were exposed to 10 Gy X-ray radiation, and CDC6 protein was assessed 1, 24, 48, and 72 h after IR exposure. **b** The protein levels of CDC6 and Ki67 were assessed in CNE2 and CNE2-R cells. **c** The protein levels of CDC6 or Ki67 were analyzed by immunohistochemical staining in tumor specimens from NPC patients with partial or complete response (PR *n* = 31 or CR *n* = 23) to radiotherapy. **d** The protein expression was scored as intensity of staining multiplied by the percentage of positive cells, and the correlation of CDC6 and Ki67 were processed for statistical analysis. **e** The CDC6 levels were assessed in the low-grade glioma tissues with/o IR and chemotherapy. These data were derived from TCGA database, and the correlations of CDC6 and tumor progression were analyzed by using statistical analysis. **f** CNE2 cells were exposed to 10 Gy IR, and the protein levels of CDC6, Rb, pRb-s780, pRb-s795, pRb-s807/811, E2F1, and E2F3 were assessed 1, 24, 48, and 72 h after IR exposure. **g** IR exposure promoted CDC6 protein stability. CNE2 and CNE2-R cells were treated with or without IR for 40 h, followed by CHX treatment. CDC6 protein was tested 0, 2, 4, 6, 8 h after CHX treatment by western blot. The half-life of CDC6 protein was calculated according to the gray-scale analysis of bands. **h** CNE2 cells were exposed to 10 Gy IR and the protein levels of APC^CDC20^ and APC^CDH1^, SCF^cyclin F^ and EMI1 were assessed 1, 24, 48, and 72 h after IR exposure. **i** The protein levels of APC^CDC20^ and APC^CDH1^, SCF^cyclin F^ and EMI1 in CNE2 and CNE2-R cells were assessed. **j** The ubiquitination status of CDC6 protein between CNE2 and CNE2-R cells was compared. pcDNA3.1-HA-Ub was transfected into CNE2 and CNE2-R cells individually. 42 h after DNA transfection, the cells were treated with 25 μM MG132 for 6 h, and then cell lysates were subjected to immunoprecipitation with anti-CDC6 antibody and immunoblotted with anti-HA antibody. **k** The expression and subcellular localization of CDC6 protein were monitored by confocal microscopy. CNE2 and CNE2-R cells were fixed and incubated with CDC6 antibody and a second antibody labeled with Alexa Fluor 488 dye. The nuclei were labeled with DAPI. **l** The nuclear and cytoplasmic parts of CNE2 or CNE2-R cells were separated, and CDC6 protein in the nuclei or cytoplasm was tested. The expression value of CDC6 protein was scored by gray-scale analysis. The expression of CDC6 in the nuclei of CNE2 or CNE2-R cells was defined by total protein minus cytoplasmic protein. **m** The phosphorylation levels of CDC6, CDK2, Cyclin A, and Cyclin E assessed in CNE2 and CNE2-R cells by western blot. *P < 0.05, ** *P* < 0.01, *** *P* < 0.001

It has been reported that IR destroyed CDC6 protein within 8 h in a p53-dependent manner [24]. However, we unexpectedly observed that IR steadily elevated CDC6 protein levels 24, 48, and 72 h after IR exposure, though the cell proliferation was retarded (Fig. 3a). Consistently, CDC6 protein levels were markedly elevated but Ki67 lowered in radioresistant CNE2-R cells compared to CNE2 cells (Fig. 3b). Similar differences were observed between radioresistant glioma U251-IR cells and their parental cells (Supplementary Figure 2D). We compared CDC6 and Ki67 protein levels in tumor tissues from NPC patients by immunohistochemistry. High CDC6 and low Ki67 levels were observed in NPC partial response (PR) tumors, vs. low CDC6 and high Ki67 levels in complete response tumors (CR, Fig. 3c). In comparison, the ratios of negative and weak CDC6-expressing tumors (IHC score 0 to 4) remarkably decreased, but the ratios of strong Ki67-expressing positive tumors (IHC score 5 to 9) significantly increased in the CR tumor tissues (Fig. 3d). From these data, we deduced that the elevation of CDC6 protein, together with the declining Ki67 (CDC6^high^Ki67^low^), probably is an important prognostic marker of cancer radioresistance.

In an effort to assess the expression levels of CDC6 in other radioresistant cancer types, we analyzed the correlations of CDC6 level and the tumor progression in the low-grade glioma with/o IR and chemotherapy. These data were derived from TCGA database. The results showed that CDC6 is positively correlated with tumor progression, and CDC6 level is much higher in the IR/chemo-treated tumors with high progression (Fig. 3e). The results validated that CDC6 is indeed a radio-specific target.

We next investigated whether acute IR-elevated CDC6 protein levels resulted from more protein synthesis or less protein degradation. E2F molecules, when activated by Rb phosphorylation, were critical transcription factors to activate CDC6 gene transcription [25]. Acute IR exposure significantly elevated the levels of unphosphorylated and phosphorylated Rb protein, but E2F1 and E2F3 proteins were not correspondingly elevated (Fig. 3f). Acute IR exposure did not distinctly increase the mRNA levels of CDC6 and five E2F family molecules (E2F1−E2F5) (Supplementary Figure 3A), and the mRNA levels of CDC6 and E2F family genes did not increase in CNE2-R cells compared to CNE2 cells (Supplementary Figure 3B). These results demonstrated that acute IR exposure elevated CDC6 protein levels but not through pRb/E2F-regulated gene transcription. IR significantly increased the half-life of CDC6 protein, and promoted its protein stability (Fig. 3g). CDC6 protein degradation with cell cycle phase is reported closely associated with ubiquitin-proteasome pathways, such as APC/C complex in G1/S phase [26] and SCF^cyclin F^ in G2/M phase [27]. We tested the levels of APC/C^CDC20^, APC/C^CDH1^ and SCF^cyclin F^ when CDC6 protein was elevated by acute or chronic IR exposure. The levels of APC/C^CDC20^ and SCF^cyclin F^ decreased while the APC/C inhibitor EMI1 increased at 72 h after radiation (Fig. 3h). These results suggested that acute IR exposure improved CDC6 protein stability partly because of the attenuation of ubiquitin-induced protein degradation.

However, the levels of E3 ubiquitin ligases did not decrease in CNE2-R cells (Fig. 3i). The ubiquitination level of CDC6 protein is similar between CNE2-R and CNE2 cells, though the protein level of CDC6 is significantly elevated in CNE2-R cells (Fig. 3j). These data suggested that the increase of CDC6 protein stability in the radioresistant cancer cells did not mainly result from ubiquitin-proteasome pathways.

The endogenous CDC6 is located inside the nuclei throughout cell cycle, whereas nuclear-cytosolic translocation is detected when CDC6 is overexpressed [28]. Protein phosphorylation by cyclin A/CDK2 is the prerequisite of CDC6 protein translocation to cytoplasm from nuclei for degradation [29]. We asked whether the insufficiency of CDC6 phosphorylation in CNE2-R cells resulted in the accumulation of CDC6 protein inside the nuclei and prevented degradation. We compared the expression and subcellular localization of CDC6 protein between CNE2-R and CNE2 cells. Higher CDC6 protein was detected in both cytoplasm and nuclei in CNE2-R cells than in CNE2 cells by confocal microscopy and biochemical methods (Fig. 3k, l). Since more CNE2-R cells were arrested in G0/G1 phase than CNE2 cells, the levels of CDK2 increased, and Cyclin A decreased in CNE2-R cells. It is well known that cyclin A phosphorylates CDC6 by direct interaction [30]. The levels of phosphorylated CDC6 (CDC6-pS54) decreased in CNE2-R cells compared to CNE2 cells (Fig. 3m). These results indicated that insufficient CDC6 protein phosphorylation decreased the nuclei-cytosol translocation of CDC6 protein, thereby attenuating CDC6 protein degradation and accumulating CDC6 protein in both nuclei and cytoplasm.

### Ectopic CDC6 overexpression impaired cell apoptosis, induced cell senescence and EMT

**Fig. 4:**
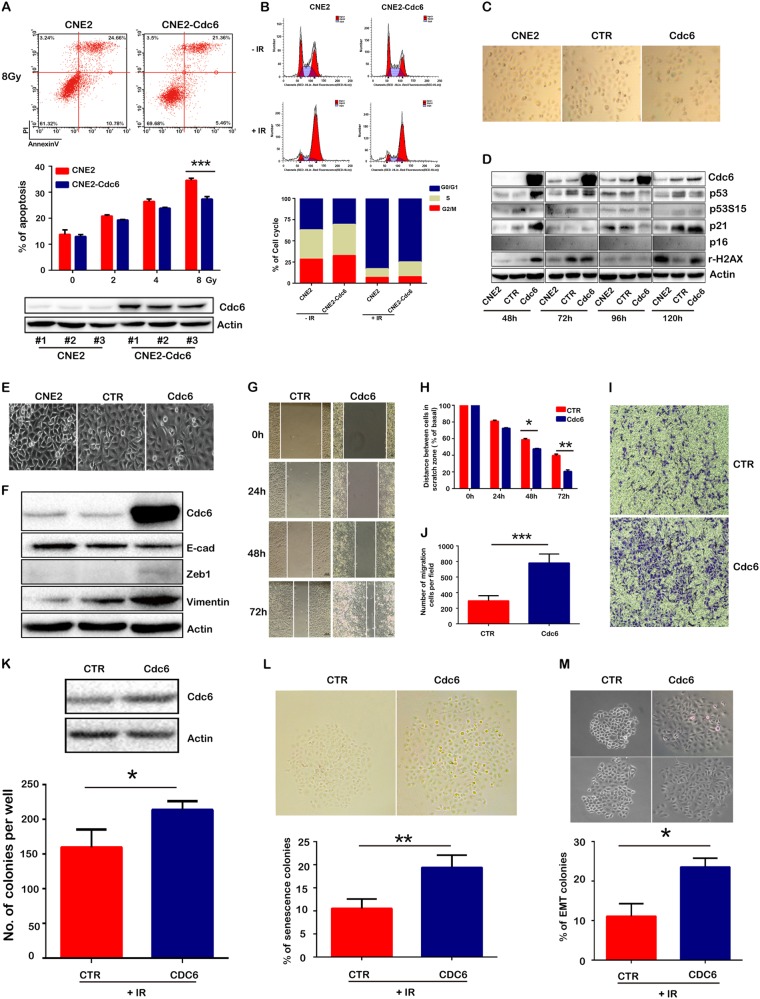
Ectopic overexpression of CDC6 promoted cell resistance to IR-induced apoptosis, altered the cell cycle distribution, and promoted senescence and EMT. **a** CNE2 cells stably overexpressing CDC6 were treated with or without IR. The apoptotic cells were stained with Annexin V-FITC/PI and detected by flow cytometry. **b** The cell cycle distribution was analyzed. **c** CNE2 cells were transiently transfected with or without CDC6 cDNA plasmid, and the cells were stained with β-galactosidase on day 5 after DNA transfection. **d** CDC6 and cell senescence-associated proteins such as p53, p53-pS15, p16, and p21 were detected by western blot 48, 72, 96, and 120 h after DNA transfection. **e** Cell morphology was observed daily after DNA transfection. **f** CDC6 and the EMT-associated proteins such as E-cadherin, Zeb1, and Vimentin were assessed by western blot. **g**, **h** Cell migration abilities were assessed by scratch wound healing assay. **i**, **j** Cell invasion abilities were assessed by transwell assay. **k** The radiosensitivity of CNE2 cells with/o CDC6 ectopic overexpression was assessed by using cell colonies formation assays when these cells were treated with IR (6 Gy). **l** CNE2 cells were transiently transfected with CDC6, and treated with IR (6 Gy). The senescent cells were stained with β-galactosidase assay, and the positive cells were quantified. The relative ratios of nonsenescent vs. senescent were calculated. **m** The clone number of epithelial or mesenchymal cells was quantified according to the morphology, and the ratios of epithelial vs. mesenchymal cells were compared in the CDC6-overexpressed CNE2 cells when treated with IR. *P < 0.05, ** *P* < 0.01, *** *P* < 0.001

In an effort to validate the roles of CDC6 in radioresistance, we artificially elevated CDC6 levels in CNE2 cells by stable transfection, and tested cell apoptosis 24 h after 2, 4, and 8 Gy IR exposure. As we expected, CDC6 overexpression impaired IR-induced cell apoptosis (34.5 vs. 26.8% at 8 Gy, Fig. 4a). CDC6 ectopic overexpression increased the cells in G0/G1 phase while decreased those in G2/M phase, which is similar to radioresistant CNE2-R cells (Fig. 1i). In contrast, CDC6 overexpression increased IR-treated cells during S phase, while decreased those in G2/M phase (Fig. 4b). Since radiosensitivity is different for cells at individual cell cycle phases, these results demonstrated that CDC6-induced cell cycle redistribution partly contributed to radiosensitivity.

CDC6 overexpression induced more cell senescence, but the cells did not exhibit typical senescence morphology (Fig. 4c). Since CNE2 cells are p16 deficient, transient transfection of CDC6 did not stimulate the expression of p16 to a visible level until 120 h, but elevated the levels of p53 at 48 and 72 h, and p21 at 48 and 120 h respectively (Fig. 4d). Therefore, in p16-deficient CNE2 cells, p53 and p21 probably take over the roles of p16, to control CDC6 overexpression-induced replicative senescence. In breast cancer MCF7 cells with p16 expression, the transient transfection of CDC6 for 48 h significantly downregulated the levels of p16, but did not influence the protein expression of p53 and p21. It had been reported that CDC6 suppressed *p16* gene transcription by an epigenetic mechanism [14]. CDC6 overexpression may induce cell senescence with time, as indicated by the elevation of p16 and p21 expression levels at 120 h after CDC6 transfection. In contrast to the control cells, CDC6 overexpression further elevated p53 and p16 levels, while decreasing the levels of p21 (Supplementary Figure 4B). These results suggested that CDC6 overexpression induced premature senescence of MCF-7 in a p16-dependent pathway.

Reportedly, mouse and human epithelial cells overexpressing CDC6 undergo EMT because CDC6 repressed *E-cadherin* transcription via the epigenetic regulatory pathways [14]. Consistent to previous reports, with the elevation of CDC6 protein, CNE2 cells exhibited typical mesenchymal morphology such as the strip shape and loose intercellular junction (Fig. 4e). The levels of E-cadherin decreased and Vimentin and Zeb1 significantly increased (Fig. 4f). Correspondingly, the cell migration and invasion capabilities of CDC6-overexpressing CNE2 cells were significantly improved, as shown in scratch wound healing assays (Fig. 4g, h) and transwell assays (Fig. 4i, j).

CDC6 ectopic overexpression decreased the radiosensitivity of CNE2 cells, as shown that the number of cell colonies significantly decreased when CDC6-overexpressed CNE2 cells were treated with IR (Fig. 4k). Since subpopulation of CNE2-R cells still showed different radiosensitivity after a long-term selection procedure, we quantified the relative ratios of nonsenescent vs. senescent and epithelial vs. mesenchymal cells in the CDC6-overexpressed CNE2 cells when treated with IR. The ratios of β-galactosidase-positive senescent cells and EMT colonies clearly increased in the CDC6-overexpressed CNE2 cells were treated with IR (Fig. 4l, m).

### CDC6 depletion enhanced IR-induced apoptosis, enhanced IR-induced senescence, and reversed EMT in CNE2-R cells

**Fig. 5:**
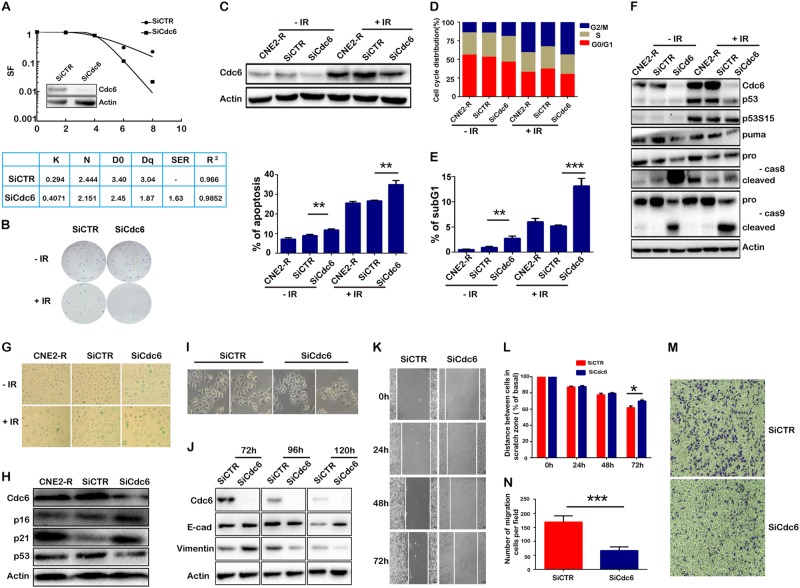
CDC6 knockdown sensitizes CNE2-R cells to X-ray radiation. CNE2-R cells were transfected with CDC6 siRNA, and cells transfected with or without nontarget siRNA were used as controls. **a** Equal numbers of cells were exposed to 0, 2, 4, 6, and 8 Gy X-ray radiation. The living cells formed cell colonies after 3 weeks, and the radiobiological parameters D0, Dq, N, and sensitizing enhancement ratio (SER) were calculated using a single-hit multitarget model of cell survival. **b** The cell colonies were stained by crystal violet when the cells were treated with 6 Gy radiation or/and CDC6 siRNA. **c** CDC6 was depleted, CDC6 protein levels were assessed by western blot, and apoptotic cells were analyzed by Annexin V-FITC/PI apoptosis detection assay. **d** The cell cycle was analyzed. **e** The subpopulation at sub-G1 represents the apoptotic cells. **f** The cell apoptosis-associated proteins were assessed by western blot. **g** CDC6 knockdown enhanced the premature senescence of CNE2 cells. The senescent cells were inoculated with β-galactosidase staining solution at PH 6, and positive cells were counted and compared (100×). **h** The cell senescence-associated proteins were assessed by western blot. **i** The comparison of morphology of CNE2 and CNE2-R cells. **j** EMT-associated proteins including E-cadherin and Vimentin were assessed by western blot. **k**, **l** Cell migration abilities were assessed by scratch wound healing assay. **m**, **n** Cell invasion abilities were assessed by transwell assay. *P < 0.05, ** *P* < 0.01, *** *P* < 0.001

Since CDC6 overexpression contributed to cancer cell radioresistance, we deduced that CDC6 depletion might sensitize cancer cells to IR-induced cell apoptosis. First, CDC6 depletion with two individual siRNA constructs significantly sensitized CNE2-R cell response to IR (Fig. 5a and Supplementary Figure 5A). The combination of CDC6 depletion and IR synergistically impaired the formation of cell colonies (Fig. 5b), and further induced cell apoptosis (Fig. 5c, e and Supplementary Figure 5B), and decreased the cells in G0/G1 phase, and increased those in G2/M phase (Fig. 5d and Supplementary Figure 5C). CDC6 depletion alone did not influence the levels of p53 and phosphorylated p53, while IR exposure increased their levels. CDC6 depletion plus IR did not further increase, but decreased the IR-increased p53 level. The combination of CDC6 depletion and IR promoted the cleavage of caspase-8 and caspase-9. The results indicated that CDC6 depletion in combination with IR exposure induced p53-independent cell apoptosis (Fig. 5f).

Since the combination of CDC6 depletion and IR could not induce the apoptosis of all radioresistant cancer cells, we tested cell senescence when CDC6 was depleted alone or in combination with IR in CNE2-R cells by β-galactosidase assay. CDC6 depletion or IR alone increased, and the combination further increased the ratios of senescent cells. CNE2-R cells exhibited typical senescence morphology in the residual CNE2-R cells after cotreatment with CDC6 depletion and IR. The cells became flat and cell size enlarged, and dark blue positive β-galactosidase staining appeared inside the cytoplasm (Fig. 5g and Supplementary Figure 5D). CDC6 depletion elevated the levels of p16 and p21, but did not increase p53 levels (Fig. 5h, f and Supplementary Figure 5E). The results demonstrated that the combination of CDC6 depletion and IR promoted CNE2-R cell to typical and mature senescence from premature senescence.

CNE2-R cells developed EMT when they obtained radioresistant features (Fig. 2c, d). We tested whether EMT would be reversed when CDC6 was depleted. CNE2-R cells changed morphology from epithelial to mesenchymal cells at day 7 after siRNA transfection (Fig. 5i and Supplementary Figure 5F). The expression of E-cadherin increased while Vimentin decreased (Fig. 5j and Supplementary Figure 5G). Correspondingly, the abilities of cell migration and invasion were significantly impaired, as shown in the scratch wound healing assays (Fig. 5k, l and Supplementary Figure 5H and I) and transwell assays (Fig. 5m, n and Supplementary Figure 5J and K).

### CDC6 knockdown sensitized IR radioresistance by enhancing IR-induced apoptosis and senescence in CNE2-R tumor xenografts

**Fig. 6:**
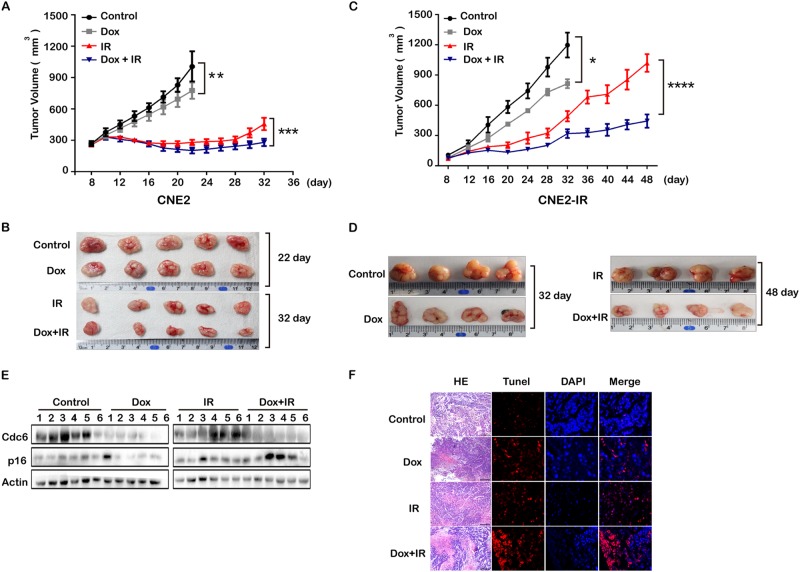
CDC6 knockdown sensitized CNE2-R tumor xenografts to IR-induced tumor regression. The CNE2-shCDC6 (**a**) or CNE2-R-shCDC6 (**c**) cells were used to generate xenografts in immune-deficient nude mice. The tumor xenografts were palpable within 2 weeks. Tumor xenografts of similar size then were treated with or without tetracycline to induce CDC6 knockdown. The CNE2-R-shRNA control and CNE2-shCDC6 xenografts were treated with or without X-ray radiation once on day 12. The tumor volumes were measured every 2 days until 32 days in CNE2-shCDC6 xenografts and every 4 days until 48 days in CNE2-R-shCDC6 xenografts. **b** Tumors of CNE2-shRNA control and CNE2-shCDC6 without IR were harvested on day 22, and tumors of CNE2-shRNA control and CNE2-shCDC6 with IR were harvested on day 32. **d** Tumors of CNE2-R-shRNA control and CNE2-R-shCDC6 without IR were harvested on day 32, and tumors of CNE2-R-shRNA control and CNE2-R-shCDC6 with IR were harvested on day 48. **e** The protein was extracted from the harvested tumors, and the protein levels of CDC6 and p16 were tested by western blot. **f** Cell apoptosis was detected in the tumor xenografts by TUNEL assay. *P < 0.05, ** *P* < 0.01, *** *P* < 0.001

The CNE2 or CNE2-R cells stably expressing inducible CDC6 shRNA were generated. The inducible CDC6 knockdown was in vitro validated by treating these cells with various doses of tetracycline (Supplementary Figure 6A and Figure 6B). Consistent for the two cell lines, CDC6 knockdown or IR exposure alone inhibited the growth of tumor xenografts, and CDC6 depletion significantly improved IR-repressed tumor growth until 32 days (Fig. 6a, b) or 48 days (Fig. 6c, d) after treatment. In an effort to elucidate whether overexpression of CDC6 is a common mechanism that causes radioresistance of NPCs, we repeated the in vivo experiments in 6-10B/6-10B-R cells, another pair of radioresistant NPC cell lines. As expected, CDC6 is overexpressed in 6-10B-R cells compared to 6-10B (Supplementary Figure 6C). The inducible CDC6 knockdown was in vitro validated by treating 6-10B or 6-10B-R cells with various doses of tetracycline (Supplementary Figure 6D and 6F). Consistent for the two cell lines, CDC6 knockdown or IR exposure alone inhibited the growth of tumor xenografts, and CDC6 depletion significantly improved IR-repressed tumor growth until 32 days after treatment (Supplementary Figure 6E and G).

The residual tumor xenografts were harvested, and CDC6 protein expression in the individual tumor specimens was assessed by western blot. CDC6 expression remarkably decreased in the tumor xenografts expressing CDC6 shRNA induced by tetracycline. The combination of CDC6 depletion and IR significantly elevated p16 levels in the tumor xenografts (Fig. 6e), which indicated that the combination of CDC6 depletion and IR significantly promoted cell senescence. TUNEL-positive apoptotic cells significantly increased with CDC6 knockdown or IR exposure, while the combination of CDC6 knockdown and IR further increased the percentage of TUNEL-positive cells (Fig. 6f). The data confirmed that CDC6 knockdown significantly sensitized cancer cells to IR-induced apoptosis and senescence.

## Discussion

The molecular mechanisms by which cancer cells develop IR resistance and promote distant metastasis remain unsettled, and biomarkers of radiosensitivity have not been identified. It is difficult for clinicians to judge which patients are suitable for radiotherapy, or predict patient response. It is urgent to identify reliable biomarkers of radiosensitivity, which will be very meaningful in clinical practice and to develop guidelines for precise and personal cancer radiotherapy.

**Fig. 7:**
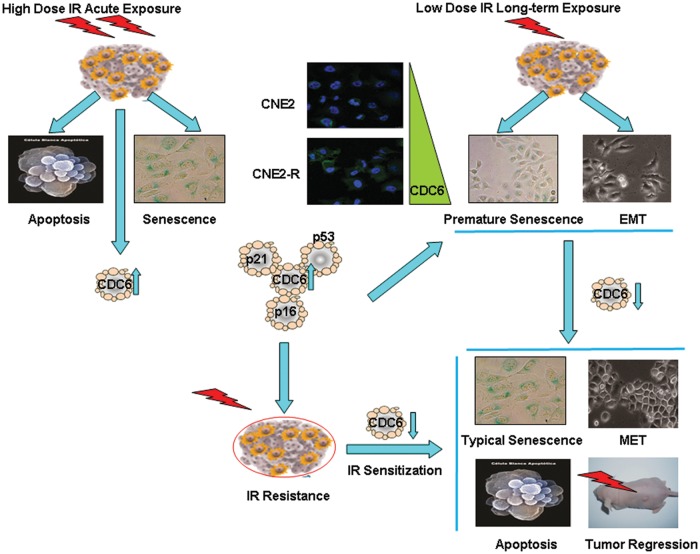
Cancer cells underwent cell apoptosis and senescence after acute exposure of high-dose IR, CDC6 protein level elevated. Cancer cells underwent premature senescence and EMT, and developed radioresistance after long-term exposure of low-dose IR. CDC6 expression significantly elevated in the radioresistant cancer cells. Ectopic elevation of CDC6 in cancer cells induced premature senescence and EMT, which are similar characteristics the radioresistant cancer cells exhibited. Conversely, the depletion of CDC6 in the radioresistant cancer cells promoted cancer cells to typical senescence from premature senescence, reversed EMT and promoted cell apoptosis. Eventually, CDC6 depletion sensitized radioresistant xenografts to IR, resulting in significant tumor regression

In this present project, CDC6 has been identified as a potential prognostic biomarker of radioresistance. Acute exposure of high-dose IR induced cell apoptosis and senescence, while cancer cells showed premature senescence and EMT, and developed radioresistance after long-term exposure of low-dose IR. The proliferation of radioresistant cancer cells was retarded, more radioresistant CNE2-R cells are arrested in G0/G1 phase, suggesting that DNA replication initiation probably has been blocked, and CDC6 is an essential molecule for DNA replication initiation [10]. It is unexpected that IR acute exposure elevated CDC6 protein levels, and higher CDC6 protein levels were detected in radioresistant CNE2-R cells. After excluding Rb/E2F-regulated gene transcription, acute IR exposure elevated CDC6 levels due to the attenuation of CDC6 protein degradation through the ubiquitin-proteasome pathways. In addition, the insufficiency of CDC6 protein phosphorylation in radioresistant cells blocked the protein nucleus-cytoplasm translocation, resulting in the subcellular accumulation of CDC6 protein. These data suggest that aberrant accumulation of CDC6 protein may play a critical role in the development of radioresistance (Fig. 7). Higher CDC6 expression has been validated in NPC tumor tissues that partially responded to IR (PR, resistant) than completely responded to IR (CR, sensitive). Since cancer cells with CDC6 overexpression showed premature senescence and retarded cell proliferation, we observed low expression of Ki67 in the PR tumor tissues.

Though a large number of CNE2-R cells were β-galactosidase positive, the cells did not exhibit typical senescent morphology. It was not expected that CNE2-R cells simultaneously exhibit both premature senescence and EMT. These phenomena are inconsistent with the current paradigm that EMT bypasses and inhibits cell senescence, since EMT-associated transcription factors such as TWIST and Zeb1 repress the cell cycle CDK inhibitors p16 and p21, and inhibit the development of senescence [31]. Radioresistant cancer cells coexhibited two phenotypes of premature senescence and EMT, which might be critical for understanding how cancer cells develop both radioresistance and promote metastasis.

CNE2 cells gained similar characteristics to radioresistant CNE2-R cells when CDC6 was ectopically overexpressed. CDC6 induced oncogene-induced replicative senescence [31] or EMT [14] in different cell models. Reportedly, CDC6 overexpression repressed the transcription of the INK4/ARF locus which encodes three important tumor suppressor genes, p16INKa, p15INKb, and ARF, all activators of the retinoblastoma pathway, an activator of p53 [32]. However, we found the transcription repression is temporary, and CDC6 overexpression initiated DNA hyper-replication, which induced DNA damage and cell senescence, resulting in the re-elevation of p16 expression (Fig. 4d). Conversely, CDC6 knockdown significantly promoted cell apoptosis, and promoted more premature cancer cells to typical senescence. Our in vivo study validated that the depletion of CDC6 resensitized radioresistant cell xenografts to IR treatment. The ectopic elevation of CDC6 induced EMT by decreasing the gene transcription of *E-cadherin* through epigenetic pathways [14]. Long-term IR exposure elevates CDC6 protein level, thereby decreasing the levels of E-cadherin and promoting cell invasion and migration. Conversely, the knockdown of CDC6 reversed EMT and inhibited cell invasion and migration by elevating the levels of E-cadherin. Therefore, CDC6 depletion sensitized cancer cells to radiation therapy, and inhibited cell invasion and migration.

In summary, this study for the first time reported cell models of IR-induced premature senescence, apoptosis resistance, and EMT, three major mechanisms of radioresistance. CDC6 was identified as a novel radioresistance switch regulating senescence, apoptosis, and EMT (Fig. 7). Our findings suggest that CDC6^high^KI67^low^ may be a new diagnostic and prognostic biomarker, and CDC6 may be a therapeutic target of cancer radiosensitization, which warrant future clinical trials.

## Materials and methods

### Cell lines and cell culture

The NPC cell lines CNE2 and 6-10B were purchased from the Xiangya Cell Bank of Central South University (Changsha, Hunan, China). The radioresistant CNE2 (CNE2-R) or 6-10B (6-10B-R) cell line was established according to previous publications [21, 33]. CNE2 and 6-10B cells were maintained in RPMI1640 medium supplemented with 10% fetal bovine serum (FBS) plus 5 mM glutamine, penicillin G (100 U/mL) and streptomycin (100 µg/mL) at 37 ℃ under 5% CO_2_. NPC HK1 cells, Glioma U251 cells, osteosarcoma U2OS cells, and breast cancer MCF-7 cells were purchased from American Type Culture Collection (ATCC, Manassas, VA, USA), and maintained in Dulbecco’s modified Eagle medium supplemented with 10% FBS and 1% penicillin/streptomycin. Radioresistant HK1-R and U251-R cells were established by a similar protocol as CNE2-R cells. The CNE2 and CNE2-R cells have been recently authenticated and confirmed no mycoplasma contamination.

### Generation of radioresistant NPC cells

CNE2 or 6-10B cells initially were exposed to 2 Gy of IR. The survived cells were passaged twice, and were exposed to additional 2 Gy of IR. The cells then were exposed to a gradually increasing IR doses (4, 6, 8, and 10 Gy, each dose twice) by following the same procedure as described above. The total IR dose is 60 Gy and the selection took over 5 months [21]. The radioresistance of CNE2-R or 6-10B-R cells was maintained with a combined and cycled IR exposure method, i.e. 10 Gy each plus 5 fractionated IR exposure (2 Gy). The radioresistance of CNE2-R or 6-10B-R cells has been validated by using a single-hit, multitarget model each exposure cycle.

### Tumor biopsy specimens

Tumor biopsy specimens were obtained from patients diagnosed with NPC in Xiangya Hospital, Central South University (Changsha, Hunan, China), and written informed consent was obtained from all participants. All tumors acquired radiotherapy after biopsy, and therapeutic effects on tumors were evaluated by CT scan 3 months after radiotherapy. Clinical efficacy was evaluated accfording to Response Evaluation Criteria Solid Tumors (RECIST1.1) [34]. In a total of 54 patients, 23 patients were assessed as complete response (CR) in which all lesions disappeared except for lymph nodes whose short axis was less than 10 mm, and 31 patients were assessed as partial response (PR) in which tumor size decreased over 30% compared to the original tumors. This study was approved by the Ethics Committee, Xiangya Hospital Central South University. After immunohistochemical staining, the expression of CDC6 and Ki67 was graded as four levels [0–3+] according to the IHC staining intensity, multiplied by the percentage of positive cells. Percentage of positive cells was graded as follows: 0 indicated no positive cells: 1+ indicated 1–25%; 2+ indicated 26–75%; and 3+ indicated more than 75%. Nine random fields (×200) were calculated for each slide, and the mean IHC scores were used to analyze the correlation of CDC6 and Ki67 protein expression by SPSS Statistics software.

### TCGA data analysis

The level of CDC6 protein expression of low-grade glioma in fragments per kilobase of transcript per million mapped reads (FPKM) was generated by TCGA. All the data were downloaded as tab-delimited files from the TCGA open-access portal (https://cancergenome.nih.gov).

### Radiosensitivity assay

The radiosensitivity of CNE2 and CNE2-R cells was assessed by cell colony-formation assay. Cells were plated in six-well plate at a density of 200, 400, 800, 1600, 3200 cells/well, and then exposed to 0, 2, 4, 6, and 8 Gy IR respectively the next day (three replicates for each dose). The surviving cells formed cell colonies 2–3 weeks after radiation, and were fixed with 2% paraformaldehyde and stained with trypan blue. The cell colonies were counted when the cells were over 50 per colony. The plating efficiency (PE) was defined as the number of formed colonies/the number of seeded cells × 100%. The survival fraction (SF) is the colonies at one IR dose divided by the number of colonies with a correction for the PE. The cell survival curve was fitted using the single-hit multitarget model of cell survival, where the probability that all *n* targets are hit is: *P*(*h*) = (1 − e^−*D*/*D*0^)^*n*^ in the Graphpad Prism 6 software. Indexes such as *D*_0_, *D*_q_, *n*, SF2, and SER (a rate of *D*0 value of CNE2 cells to CNE2-R) were calculated to evaluate cell radiosensitivity.

### Animal experiments

The animal experiment protocol was approved by the Ethics Committee of Xiangya Hospital Central South University. Tetracycline-inducible CDC6 knockdown cell lines CNE-2-shCDC6, CNE2-R-shCDC6, 6-10B-shCDC6 or 6-10B-R-shCDC6 cells were generated and used to induce tumor xenografts in the nude mice. The cells were injected (5×10^6^/100 μL) into the subcutaneous tissue of the left flank region of 6-week-old immune-deficient nude mice (BALB/C-nu/nu, SLAC Laboratory, Shanghai, China). Transplanted tumor xenografts attained 100 mm^3^ 3 weeks after cell incubation, and mice were randomly divided into four treatment groups (*n* = 6/group): Control, CDC6 knockdown, IR (6 Gy) and CDC6 knockdown+ IR. CDC6 knockdown was induced in animals by feeding with tetracycline-containing diets starting from day 0 (Research Diets, New Brunswick, NJ, USA) in the CDC6 knockdown and CDC6 knockdown+ IR groups, while the mice in the control and IR groups were maintained with routine diets. The tumors in the IR and CDC6 knockdown+ IR groups were treated with 6 Gy X-radiation once at day 12 when tumor size reached about 200 mm^3^. Tumor sizes were measured every 4 days starting from day 8, and volume (*V*) was calculated using the following formula: *V* = *ab*^2^/2 (*a*, the long diameter and b, the short diameter). The mice were sacrificed when tumor volume reached 1100 mm^3^ or the 44 days after treatments. The tumor xenografts were collected and divided in half. Half the tumor was stored in liquid nitrogen and used for the analysis of CDC6 and p16 protein expression by western blotting, and the other half was used for apoptosis detection by TUNEL staining.

### Statistical analysis

Data are presented as the mean ± SD. Statistical analysis was performed by a two-tailed unpaired *t* test between two groups. For the tumor xenograft experiment, groups were compared by two-way ANOVA. The two-tailed Pearson correlation between CDC6 expression and the responses to radiotherapy of NPC patients, and the expression of Ki67, was calculated using SPSS Statistics software (IBM, Armonk, NY, USA), and *P* values were determined by two-tailed Fisher’s exact test. Results were considered significant for *P < 0.05, **P < 0.01, ***P < 0.001.

## Electronic supplementary material

Supplementary Data

Supplementary Figure 1

Supplementary Figure 2

Supplementary Figure 3

Supplementary Figure 4

Supplementary Figure 5

Supplementary Figure 6

## References

[1] Wei WI, Sham JS (2005). Nasopharyngeal carcinoma. Lancet.

[2] Lee AW, Sze WM, Au JS, Leung SF, Leung TW, Chua DT (2005). Treatment results for nasopharyngeal carcinoma in the modern era: the Hong Kong experience. Int J Radiat Oncol Biol Phys.

[3] Suarez C, Rodrigo JP, Rinaldo A, Langendijk JA, Shaha AR, Ferlito A (2010). Current treatment options for recurrent nasopharyngeal cancer. Eur Arch Oto-Rhino-Laryngol.

[4] Chen W, Hu GH (2015). Biomarkers for enhancing the radiosensitivity of nasopharyngeal carcinoma. Cancer Biol Med.

[5] Raleigh DR, Haas-Kogan DA (2013). Molecular targets and mechanisms of radiosensitization using DNA damage response pathways. Future Oncol.

[6] Kurrey NK, Jalgaonkar SP, Joglekar AV, Ghanate AD, Chaskar PD, Doiphode RY (2009). Snail and slug mediate radioresistance and chemoresistance by antagonizing p53-mediated apoptosis and acquiring a stem-like phenotype in ovarian cancer cells. Stem Cells.

[7] Lee M, Lee JS (2014). Exploiting tumor cell senescence in anticancer therapy. BMB Rep.

[8] Chang BD, Swift ME, Shen M, Fang J, Broude EV, Roninson IB (2002). Molecular determinants of terminal growth arrest induced in tumor cells by a chemotherapeutic agent. Proc Natl Acad Sci USA.

[9] Madani I, De Neve W, Mareel M (2008). Does ionizing radiation stimulate cancer invasion and metastasis?. Bull Cancer.

[10] Borlado LR, Mendez J (2008). CDC6: from DNA replication to cell cycle checkpoints and oncogenesis. Carcinogenesis.

[11] Liu Y, Hock JM, Van Beneden RJ, Li X (2014). Aberrant overexpression of FOXM1 transcription factor plays a critical role in lung carcinogenesis induced by low doses of arsenic. Mol Carcinog.

[12] Young A, Berry R, Holloway AF, Blackburn NB, Dickinson JL, Skala M (2014). RNA-seq profiling of a radiation resistant and radiation sensitive prostate cancer cell line highlights opposing regulation of DNA repair and targets for radiosensitization. BMC Cancer.

[13] Bartkova J, Rezaei N, Liontos M, Karakaidos P, Kletsas D, Issaeva N (2006). Oncogene-induced senescence is part of the tumorigenesis barrier imposed by DNA damage checkpoints. Nature.

[14] Sideridou M, Zakopoulou R, Evangelou K, Liontos M, Kotsinas A, Rampakakis E (2011). Cdc6 expression represses E-cadherin transcription and activates adjacent replication origins. J Cell Biol.

[15] Feng D, Tu Z, Wu W, Liang C (2003). Inhibiting the expression of DNA replication-initiation proteins induces apoptosis in human cancer cells. Cancer Res.

[16] Liu Y, Hock JM, Sullivan C, Fang G, Cox AJ, Davis KT (2010). Activation of the p38 MAPK/Akt/ERK1/2 signal pathways is required for the protein stabilization of CDC6 and cyclin D1 in low-dose arsenite-induced cell proliferation. J Cell Biochem.

[17] Liu Y, Gong Z, Sun L, Li X (2014). FOXM1 and androgen receptor co-regulate CDC6 gene transcription and DNA replication in prostate cancer cells. Biochim Biophys Acta.

[18] Hampel B, Malisan F, Niederegger H, Testi R, Jansen-Durr P (2004). Differential regulation of apoptotic cell death in senescent human cells. Exp Gerontol.

[19] Rufini A, Tucci P, Celardo I, Melino G (2013). Senescence and aging: the critical roles of p53. Oncogene.

[20] Kuilman T, Michaloglou C, Mooi WJ, Peeper DS (2010). The essence of senescence. Genes Dev.

[21] Li G, Liu Y, Su Z, Ren S, Zhu G, Tian Y (2013). MicroRNA-324-3p regulates nasopharyngeal carcinoma radioresistance by directly targeting WNT2B. Eur J Cancer.

[22] Pawlik TM, Keyomarsi K (2004). Role of cell cycle in mediating sensitivity to radiotherapy. Int J Radiat Oncol Biol Phys.

[23] Chang JC (2016). Cancer stem cells: role in tumor growth, recurrence, metastasis, and treatment resistance. Medicine.

[24] Duursma A, Agami R (2005). p53-dependent regulation of Cdc6 protein stability controls cellular proliferation. Mol Cell Biol.

[25] Hateboer G, Wobst A, Petersen BO, Le Cam L, Vigo E, Sardet C (1998). Cell cycle-regulated expression of mammalian CDC6 is dependent on E2F. Mol Cell Biol.

[26] Clijsters L, Ogink J, Wolthuis R (2013). The spindle checkpoint, APC/C(Cdc20), and APC/C(Cdh1) play distinct roles in connecting mitosis to S phase. J Cell Biol.

[27] Walter D, Hoffmann S, Komseli ES, Rappsilber J, Gorgoulis V, Sorensen CS (2016). SCF(Cyclin F)-dependent degradation of CDC6 suppresses DNA re-replication. Nat Commun.

[28] Alexandrow MG, Hamlin JL (2004). Cdc6 chromatin affinity is unaffected by serine-54 phosphorylation, S-phase progression, and overexpression of cyclin A. Mol Cell Biol.

[29] Petersen BO, Wagener C, Marinoni F, Kramer ER, Melixetian M, Lazzerini Denchi E (2000). Cell cycle- and cell growth-regulated proteolysis of mammalian CDC6 is dependent on APC-CDH1. Genes Dev.

[30] Di Micco R, Fumagalli M, Cicalese A, Piccinin S, Gasparini P, Luise C (2006). Oncogene-induced senescence is a DNA damage response triggered by DNA hyper-replication. Nature.

[31] Smit MA, Peeper DS (2010). Epithelial−mesenchymal transition and senescence: two cancer-related processes are crossing paths. Aging.

[32] Gonzalez S, Klatt P, Delgado S, Conde E, Lopez-Rios F, Sanchez-Cespedes M (2006). Oncogenic activity of Cdc6 through repression of the INK4/ARF locus. Nature.

[33] Su Z, Li G, Liu C, Ren S, Tian Y, Liu Y (2016). Ionizing radiation promotes advanced malignant traits in nasopharyngeal carcinoma via activation of epithelial-mesenchymal transition and the cancer stem cell phenotype. Oncol Rep.

[34] Eisenhauer EA, Therasse P, Bogaerts J, Schwartz LH, Sargent D, Ford R (2009). New response evaluation criteria in solid tumours: revised RECIST guideline (version 1.1). Eur J Cancer.

