# Supplementary material for: Radiation-promoted CDC6 protein stability contributes to radioresistance by regulating senescence and epithelial to mesenchymal transition
Source: Oncogene. 2018 Aug 29;38(4):549–63. doi: 10.1038/s41388-018-0460-4 (PMC6345673; doi:10.1038/s41388-018-0460-4)
Supplement: Supplementary file 1 — Supplementary Data [file 41388_2018_460_MOESM1_ESM.doc]

**Supplementary Materials and Methods**

**Immunohistochemistry**

Paraffin-embedded specimens were sectioned, deparaffinized and rehydrated using xylene and decreasing grades of ethanol. The sec­tions were boiled for antigen retrieval, and immersed in 3% H2O2 for 10 min to quench endogenous peroxidase. Sections were then blocked with 10% serum for 1 h to reduce nonspecific staining, and then incubated overnight at 4°C with primary antibodies. Afterward, the sections were rinsed and then coated with an HRP-conjugated second antibody and incubated at 37°C for 1 h. DAB was used to visualize immunoreaction sites. Sections were counterstained with hematoxylin and mounted on glass slides.

**Plasmids, siRNAs and shRNAs**

CDC6 cDNA in the pCMV-XL5 vector was purchased from Origene (Rockville, MD, USA). ON-Target plus SMART pool small interfering RNA (siRNA) for the targets of CDC6, and non-targeting siRNA were purchased from Dharmacon Inc. (Lafayette, CO, USA) via GE Life Sciences, China. pLV-E2F1a-CDC6-IRES-Bsd or pLVET-tTR-KRAB-shRNA-CDC6 was constructed by the following subcloning strategies: the CDC6 cDNA fragment was cut with *SmalI* and *BamHI* from pCMV-XL5-CDC6 vector, and inserted into pLV-EF1a-MCS-IRES-Bsd (Biosetta, San Diego, CA, USA). The nucleotide sequence of shRNA-CDC6 and shRNA-CTR was designed as shown in the supplemental data. shRNA-CDC6 or shRNA-CTR was subcloned into pLVTHM vector by *MluI* and *ClaI*, and then inserted into the doxycycline inducible plasmid vector pLVET-tTR-KRAB (Addgene, Cambridge, MA, USA) by *MscI* and *FspI*.

**Transient transfection assay**

pCMV-XL5-CDC6 was transfected to CNE2 cells using a Lipofectamin 2000 Transfection Kit (Life Technology, Carisbad, CA, USA). siRNA transfection was performed using Dharma FECT Transfection Reagent (Thermo Fisher Scientific, Wilmington, DE, USA). CNE2 or MCF-7 cells were plated in 6-well plates at the dentistry of 3×10^5^/well and transfected with 2 ug pCMV-XL5-CDC6 or pCMV-XL5 control. CNE2-R cells were transfected with 50 nM siRNA-CDC6 or siRNA-control. Cell senescence, migration and invasion were tested 48h after transfection. To evaluate the combined effects of CDC6 knockdown and radiation, CNE2-R cells were transfected with CDC6 siRNA, followed by 10 Gy radiation 48 hours after transfection. The cells were harvested to analyze cell apoptosis, senescence and cell cycle 48h after radiation.

**Lentiviral package and stable transduction**

CDC6-expressing lentivirus or sh-CDC6-encoding lentivirus was produced by co-transfecting the plasmids of pLV-EF1a-CDC6-IRES-Bsd or pLVET-tTR-KRAB-shRNA-CDC6, the packaging plasmids (Gag-Pol and Rev) and the envelope plasmid (VSV-G) to HEK-293T cells following the standard manuals of lentivirus production. CNE2 cells were infected with high titer CDC6-expressing lentivirus. The infected cells were selected by 1 µg/ml Bsd for 3 continuous weeks. Immunoblot analysis was used to assess the protein level of CDC6 in CNE2 cells. CNE2-IR cells were infected with shRNA-CDC6-encoding lentivirus. Since the plasmid of PLVET-tTR-KRAB-shRNA-Cdc6 expresses GFP, the lentivirus-infected cells with green-fluorescence were sorted by flow cytometry, and the inducible CDC6 knockdown was confirmed by western blot after the cells were treated with stepwise increasing doses of doxycycline.

**Western blot**

Cells were lysed using the RIPA lysis buffer (Beyotime, Shanghai, China). Equal amounts of proteins were separated by SDS-PAGE and transferred to polyvinylidene difluoride membranes (Bio-Rad Laboratories, Shanghai, China). The membranes were blocked in 5% milk with 0.1% Tween 20 TBS at room temperature for 1 h, and then incubated with primary antibodies respectively overnight at 4°C and then the membranes were incubated with HRP-conjugated secondary antibodies and detected using the ECL Plus kit (Thermo Fisher Scientific, Shanghai, China).

***In vitro* ubiquitylation assay**

CNE2 and CNE2-R cells were transfected with pcDNA3.1/HA Ubiquitin, and cells were treated with 25 uM MG132 for 6h. Cells were lysed in RIPA, and equal amounts of total protein were incubated with antibody CDC6 and protein A/G PLUS- Agarose (sc-2003) following the manufacturer’s protocol. Immunoprecipitates were subjected to polyacrylamide SDS-PAGE, and ubiquitination of CDC6 was detected by anti-HA antibody.

**Cell cycle analysis**

The cells were fixed in 70% anhydrous ethanol at 4℃ overnight. The cells were gently washed twice with PBS and then incubated with RNase at 37°C for 1 h, followed by incubation with 100 μg/ml propidium iodide (PI) at room temperature for 30 min. The cell cycle profiles were analyzed by flow cytometry, and the data were analyzed using modFit LT software.

**Apoptosis detection assay**

Cells were harvested after treatment and gently washed twice with PBS. The cells were incubated with Annexin V-FITC and propidium iodide (PI) from the Apoptosis Detection Kit (KeyGEN BioTECH, Nanjing, China) at room temperature for 15 minutes. The percentage of positive cells was assessed by the flow cytometry.

**Senescence-associated β-Galactosidase assay**

The senescent cells were fixed and stained with a β-Galactosidase staining kit (CST). The experimental procedure followed the manufacturer’s protocol. Images were acquired using a DMIL LED inverted microscope (Leica) with a 20×/0.4-NA objective. Cells with β-Galactosidase positive staining and typical senescence morphology were analyzed by Image Pro Plus v3.0 software.

**Scratch wound-healing assays**

CNE2 cells were transfected with or without pCMV-XL5-CDC6 plasmid DNA, and pCMV-XL5 vector was used as the control. CNE2-R cells were transfected with siRNA-CDC6, and the non-targeting siRNA was used as the control. CNE2 cells and CNE2-R cells were seeded on 6-well plates, and a scratch wound was performed using a sterile 200 µL pipette tip when cell confluence was up to 90%. Phase-contrast images were taken at the starting time point (0 h) and at various time points up to 72 h by inverted microscopy (DMIL LED, Leica). Data were presented as percentages of the remaining gap distance relative to the initial gap distance, and data were averaged from three independent measurements. The corresponding SD is also reported.

**Cell invasion transwell assay**

CNE2 and CNE2-R cells were seeded on top of matrigel–coated transwells (BD) at 20,000 cells per well in 24-well plates with serum-free medium. The transwell inserts were embedded into complete culture medium, and cells were allowed to invade for 24h. At the end of the assay, the cells on the top side of the well were scraped, and the invading cells on the bottom side of the well were stained with 1% crystal violet, photographed, and counted.  Three replicates were used for each cell line. Data from three independent measurements were averaged, and the corresponding SD is reported.

**Cell immunofluorescence**

For the analysis of CDC6 subcellular localization, CNE2 and CNE2-R cells grown on a coverslip were washed with PBS and fixed with 4% paraformalinhydye for 15 min. After washing twice with PBS, the cells were permeabilized with 0.2% Triton X-100 for 20 min and blocked with 5% BSA for 30 min at room temperature. Cells were then incubated with anti-CDC6 antibody (1:200 dilution in 5% BSA ) overnight at 4℃, washed with PBST three times, and incubated with Alexa Fluor® 488 Donkey Anti-Goat IgG (1:2000 dilutions; Invitrogen) for 1 h at room temperature. After washing with PBST three times, cells were further stained with DAPI for 10 min. All images were collected by confocal laser scanning microscope (SP8, Leica) equipped with a Plapon 60× 1.42 NA oil lens objective.

**TUNEL staining**

TUNEL staining was performed using a TUNEL Apoptosis Assay Kit (Beyotime, Shanghai, China) according to the manufacturer’s instructions. In brief, formalin-fixed paraffin-embedded sections from the transplanted tumors were deparaffinized by xylene and rehydrated in a graded series of ethanol aqueous solutions. After being washed twice with PBS, the sections were incubated with DNase-free protease K (20ug/ml) at 37℃ for 30 min. The TUNEL reaction mixture (5 µl of labeling solution and 45 µl of enzyme solution) was added to the specimens and incubated at 37℃ for 1 h after being rinsed with PBS twice. The mixture was then removed and the sections were washed with PBS three times, and then incubated with DAPI to label the nuclei.

**Supplementary Figure Legends**

**Supplementary Figure 1.** IR exposure induced cell apoptosis and senescence in U2OS cells. U2OS cells were exposed to 6 Gy IR, cell apoptosis and senescence were tested and the expression of associated proteins was assessed 1，24, 48 and 72 hour after IR exposure. Cells without IR exposure were used as controls. A. cell apoptosis was detected by Annexin V-FITC/PI staining and the early or late apoptotic cells were analyzed by flow cytometry. B. Cell senescence was detected by senescence-associated β-Galactosidase assay. Senescent cells with β-Galactosidase positive staining and enlarged cell size were counted for statistical analysis. C. IR-exposed cells were harvested and the proteins were extracted 1，24, 48 and 72 hours after IR exposure. Protein expression of CDC6, γH2AX (DNA damage), p53, p53-s15(cell apoptosis and senescence), p16 and p21(cell senescence) and BCL-XL and PUMA (apoptosis) was detected by western blot.

**Supplementary Figure 2.** A. HK1-R cells were induced when HK1 cells were exposed to low dose of radiation. HK1-R cells exhibited typical EMT after low-dose radiation exposure. A. The morphology of HK1-R and HK1. B. The protein levels of CDC6 and EMT-associated proteins such as E-cadherin and Vimentin were assessed by western blot. C. More sub-population of cancer stem cells (CD44+CD24+) were detected in CNE2-R cells than CNE2 cells. D. U251-R cells were induced when HK1 cells were exposed to low dose radiation. The protein levels of CDC6 and Ki67 were assessed in U251 and U251-R cells.

**Supplementary Figure 3.** Acute IR exposure did not change the mRNA levels of CDC6 and five E2F family molecules. CNE2 cells were exposed to 10 Gy IR and mRNA levels were assessed 1, 24, 48 and 72 hours after IR exposure. B. The mRNA level of CDC6 and five E2F family molecules was assessed in CNE2-R and CNE2 cells. C. Protein levels of CDC6 in CNE2 cells were assessed within 12 hours after acute IR exposure.

**Supplementary Figure 4.**  Ectopic overexpression of CDC6 promoted senescence in MCF7 cells. A. MCF7 cells were transiently transfected with or without CDC6 cDNA plasmids, and the cells were stained with β-galactosidase on day 5 after DNA transfection. B. CDC6 and cell senescence-associated proteins such as p53, p53-pS15, p16 and p21 were assessed by western blot at 48, 72, 96 and 120 hours after DNA transfection. C. CDC6 was depleted by siRNA in MCF7 cells, and the cells in sub-G1, G0/G1, S or G2/M phase was analyzed by flow cytometry. D. CDC6 was depleted by siRNA in MCF7 cells, and the protein levels of apoptosis-associated molecules, p53 and phosphorylated p53 were assessed in MCF7 cells with or without CDC6 knockdown and radiation exposure.

**Supplementary Figure 5.** CDC6 knockdown sensitizes CNE2-R cells to X-ray radiation. CNE2-R cells were transfected with CDC6 siRNA, and cells transfected with or without non-target siRNA were used as controls. **A.** Equal numbers of cells were exposed to 0, 2, 4, 6 and 8 Gy X-ray radiation. The living cells formed cell colonies after three weeks, and the radiobiological parameters D0, Dq, N, and sensitizing enhancement ratio (SER) were calculated using a single-hit multi-target model of cell survival. **B.** CDC6 was depleted, CDC6 protein levels were assessed by western blot, and apoptotic cells were analyzed by Annexin V-FITC/PI apoptosis detection assay. **C.** The cell cycle was analyzed. **D.** CDC6 knockdown enhanced the premature senescence of CNE2 cells. The senescent cells were inoculated with β-galactosidase staining solution at PH 6, and positive cells were counted and compared (100×). **E.** The cell senescence-associated proteins were assessed by western blot. **F.** The comparison of morphology of CNE2-R cells when CDC6 was depleted by siRNAs. **G.** EMT-associated proteins including E-cadherin and Vimentin were assessd by western blot. **H.** and **I**. Cell migration abilities were assessed by scratch wound healing assay. **J.** and **K.** Cell invasion abilities were assessed by transwell assay.

**Supplementary Figure 6.** The tetracycline-inducible stable cell lines CNE2-shCDC6, CNE2-R-shCDC6, 6-10B-shCDC6 and 6-10B-R-shCDC6 were generated. (**A.**) CDC6 protein levels decreased with the doses of tetracycline in CNE2-shCDC6 cells. (**B.**) The lentiviral vector encoding a GFP gene and the green fluorescent cells represent the virus-infected cells. CDC6 protein levels decreased with the doses of tetracycline in CNE2-R-shCDC6 cells. (**C.**) The protein levels of CDC6 were compared between 6-10B and 6-10B-R cells. (**D.**) and **(F.)** The tetracycline-inducible stable cell lines 6-10B-shCDC6 and 6-10B-R-shCDC6, and CDC6 knockdown was validated by western blot when the cells were treated with stepwise increasing doses of doxycycline. **(E.)** and **(G.)** 6-10B-shCDC6 or 6-10B-R-shCDC6 cells were used to generate tumor xenografts in immune-deficient nude mice. The tumor xenografts were palpable within two weeks. Tumor xenografts of similar size then were treated with or without doxycycline to induce CDC6 knockdown. The 6-10B-shRNA **(E.)** and 6-10B-R-shCDC6 **(G.)** xenografts were treated with or without X-ray radiation once on day 12. The tumor volumes were measured every 4 days until 32 days.

**Supplementary Table 1**

**Primers used in the project**

| Uses | Primer Name | Sequence |
| --- | --- | --- |
| Real-time PCR | CDC6-F | GGGAATCAGAGGCTCAGAAG |
|  | CDC6-R | CACTGGATGTTTGCAGGAGA |
|  | E2F1-F | ACGCTATGAGACCTCACTGAA |
|  | E2F1-R | TCCTGGGTCAACCCCTCAAG |
|  | E2F2-F | CGTCCCTGAGTTCCCAACC |
|  | E2F2-R | GCGAAGTGTCATACCGAGTCTT |
|  | E2F3-F | AGAAAGCGGTCATCAGTACCT |
|  | E2F3-R | TGGACTTCGTAGTGCAGCTCT |
|  | E2F4-F | CACCACCAAGTTCGTGTCCC |
|  | E2F4-R | GCGTACAGCTAGGGTGTCA |
|  | E2F5-F | ATGTCTTCTGACGTGTTTCCTC |
|  | E2F5-R | CGGGGTAGGAGAAAGCCTT |
| Plv-EF1a-MCS-  IRES-CDC6 | CDC6-F-Sma1 | CCCCCGGGATGCCTCAAACCCGATCCC |
|  | CDC6-R-BamH1 | CGGGATCCTTAAGGCAATCCAGTAGCTAAGA |
| shRNA-CDC6 | sh-Cdc6-F | cgcgtccccATTTGGAGGACACTGGTTAAATTCAAG  AGATTTAACCAGTGTCCTCCAAATTTTTTTggaaa |
|  | sh-Cdc6-R | cgatttccAAAAAAATTTGGAGGACACTGGTTAAATC  TCTTGAATTTAACCAGTGTCCTCCAAATgggga |
| ShRNA-CTR | sh-CTR-F | cgcgtccccCCTAAGGTTAAGTCGCCCTCGCTCGAG  CGAGGGCGACTTAACCTTAGGTTTTTggaaat |
|  | sh-CTR-R | cgatttccAAAAACCTAAGGTTAAGTCGCCCTCGC  TCGAGCGAGGGCGACTTAACCTTAGGgggga |

**Supplementary Table 2**

**Antibodies used in the project**

| **Antibody** | **Company** | **Catalog No.** |
| --- | --- | --- |
| CDC6 | Santa Cruz Biotechnology | sc-9964 |
| P53 | Santa Cruz Biotechnology | sc-53395 |
| P53-Ser15 | Cell Signaling Technology | 9284 |
| PUMA | Cell Signaling Technology | 4976S |
| BCL-XL | Santa Cruz Biotechnology | sc-8392 |
| P21 | Cell Signaling Technology | 2947 |
| P16 | Santa Cruz Biotechnology | SC-468 |
| Rb | Cell Signaling Technology | 9309 |
| E-cadherin | Santa Cruz Biotechnology | sc-21791 |
| N-cadherin | Cell Signaling Technology | 13116 |
| Vimentin | Abcam | ab8978 |
| Zeb1 | Santa Cruz Biotechnology | sc-10572X |
| Twist | Santa Cruz Biotechnology | sc-81417 |
| Ki67 | ABclonal | A2094 |
| Rb-phospho-(Ser807/811) | Cell Signaling Technology | 8516 |
| Rb-phospho-(Ser795) | Cell Signaling Technology | 9301 |
| Rb-phospho-(Ser780) | Cell Signaling Technology | 8180 |
| E2F3 | Santa Cruz Biotechnology | sc-28308 |
| E2F1 | Santa Cruz Biotechnology | sc-137059 |
| CyclinF | Santa Cruz Biotechnology | sc-515207 |
| Emi1 | Santa Cruz Biotechnology | sc-365212 |
| fzr | Santa Cruz Biotechnology | sc-56312 |
| p55CDC | Santa Cruz Biotechnology | sc-13162 |
| HA-probe | Santa Cruz Biotechnology | sc-805 |
| Cdc6 pS54 | Abcam | Ab75809 |
| CyclinA | Santa Cruz Biotechnology | sc-136253 |
| CyclinE | Santa Cruz Biotechnology | sc-377100 |
| Cdk2 | Santa Cruz Biotechnology | sc-390283 |
| Caspase8 | Santa Cruz Biotechnology | sc-81657 |
| Caspase9 | Santa Cruz Biotechnology | sc-81663 |
| Actin | Santa Cruz Biotechnology | sc-58673 |
| Tublin | Santa Cruz Biotechnology | sc-69969 |
