# Supplementary figures and images for: Radiation-promoted CDC6 protein stability contributes to radioresistance by regulating senescence and epithelial to mesenchymal transition
Source: Oncogene. 2018 Aug 29;38(4):549–63. doi: 10.1038/s41388-018-0460-4 (PMC6345673; doi:10.1038/s41388-018-0460-4)

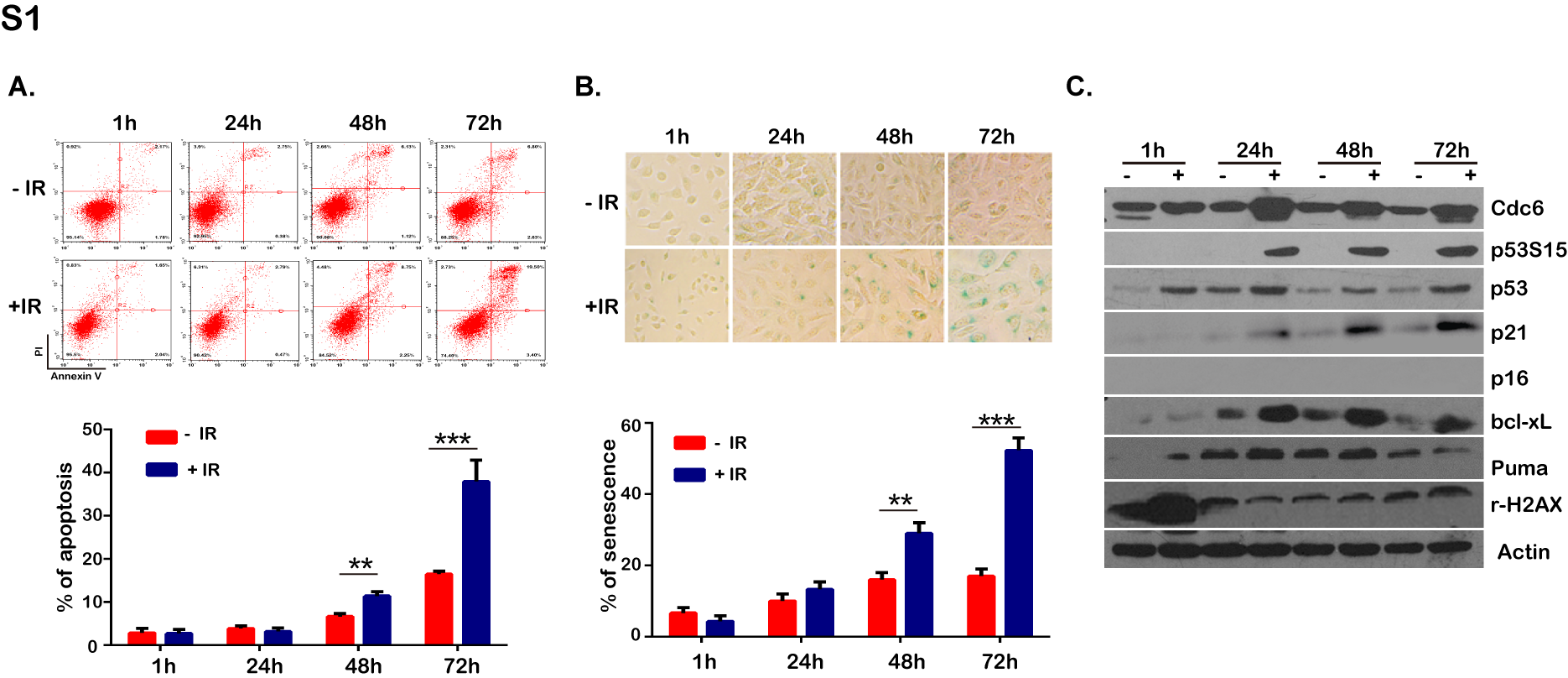

Supplement: Supplementary file 2 — Supplementary Figure 1 [file 41388_2018_460_MOESM2_ESM.tif]

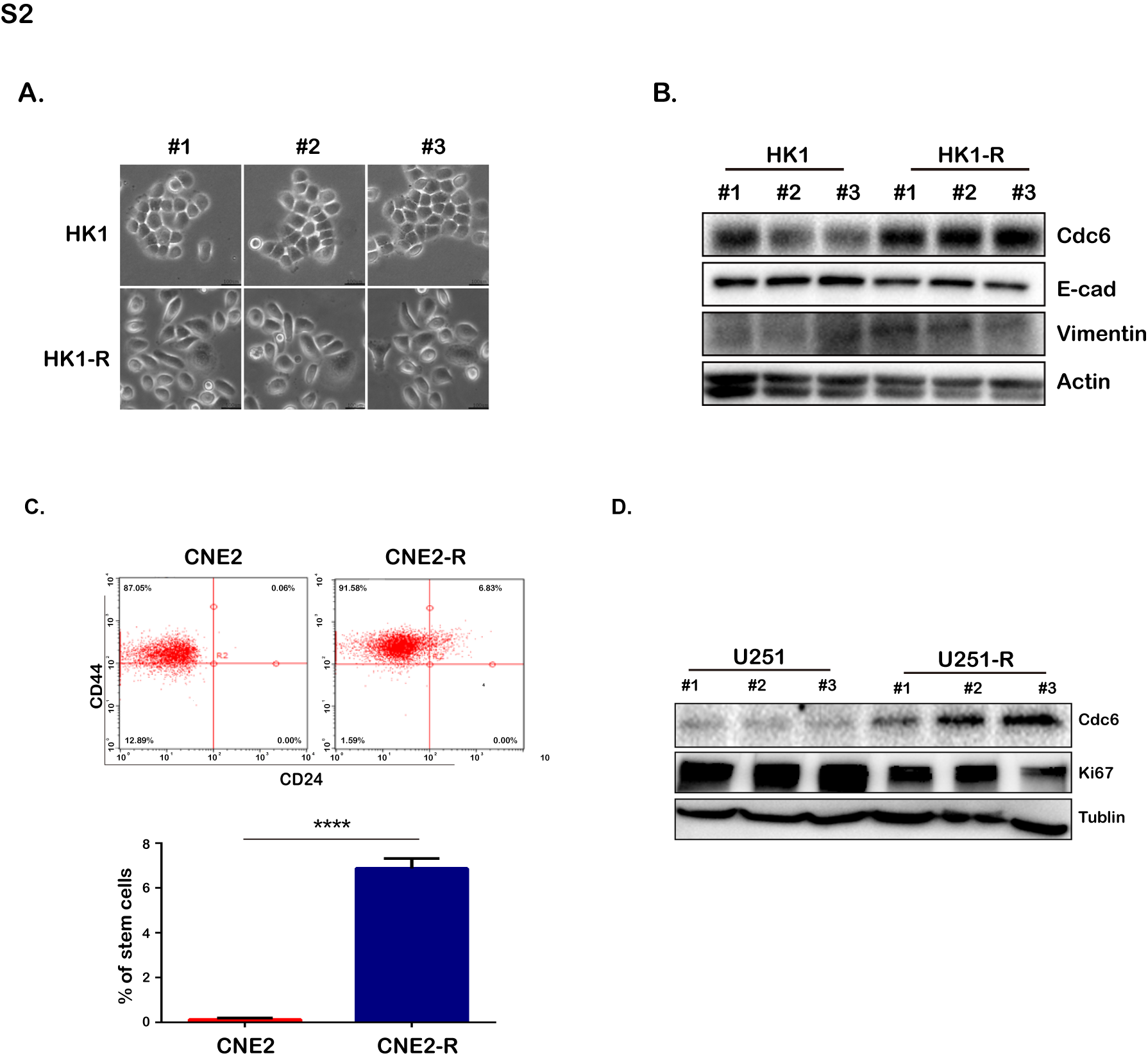

Supplement: Supplementary file 3 — Supplementary Figure 2 [file 41388_2018_460_MOESM3_ESM.tif]

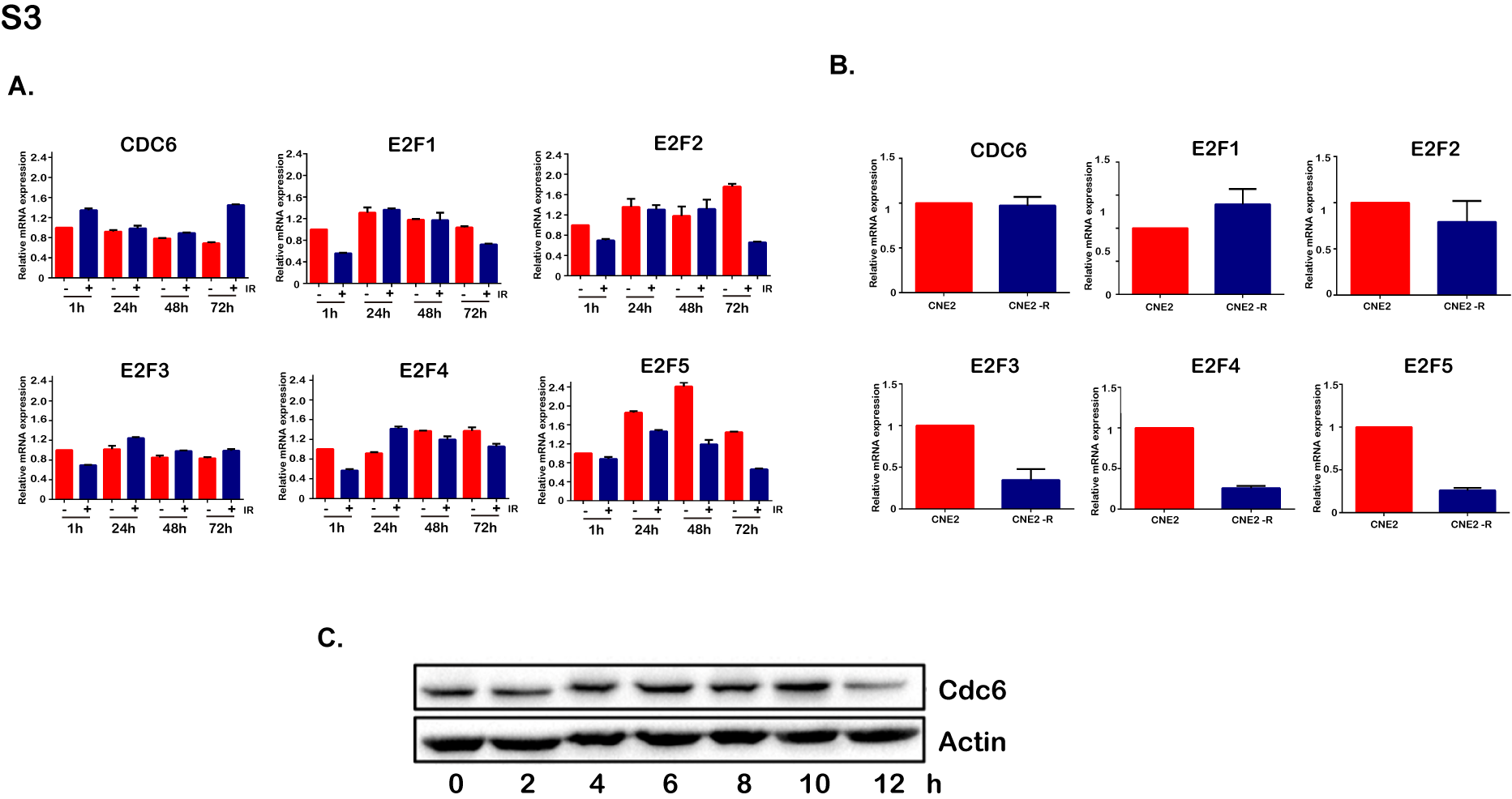

Supplement: Supplementary file 4 — Supplementary Figure 3 [file 41388_2018_460_MOESM4_ESM.tif]

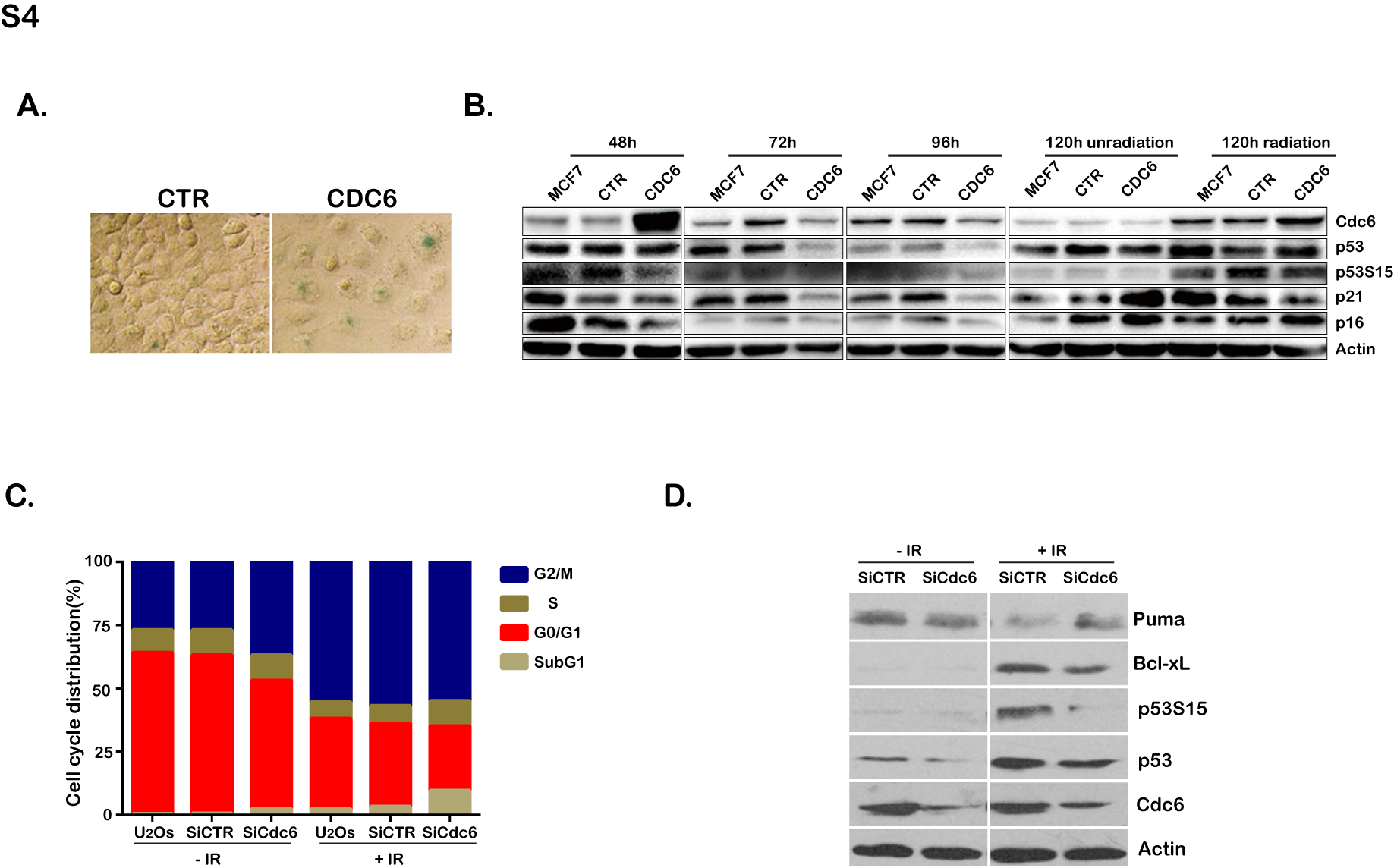

Supplement: Supplementary file 5 — Supplementary Figure 4 [file 41388_2018_460_MOESM5_ESM.tif]

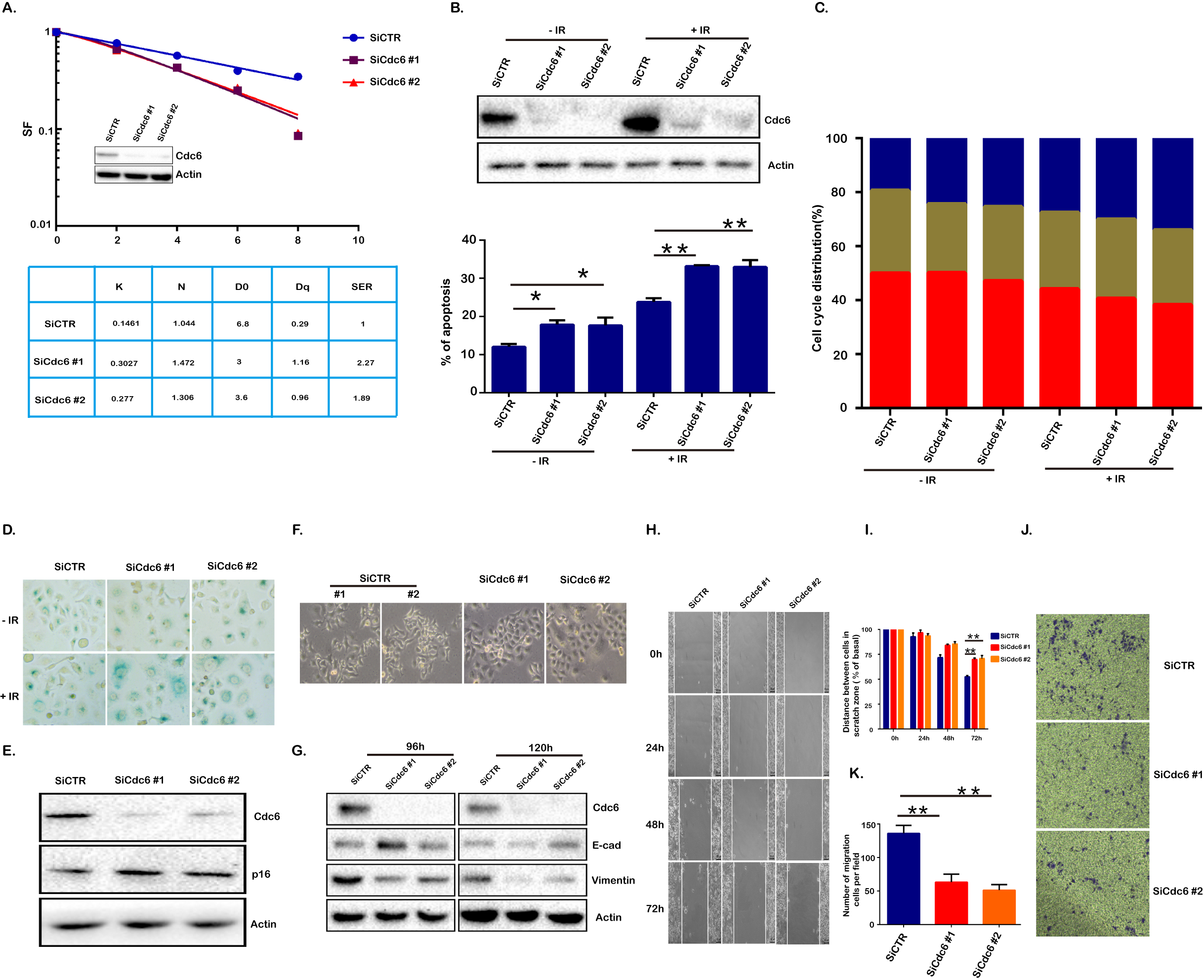

Supplement: Supplementary file 6 — Supplementary Figure 5 [file 41388_2018_460_MOESM6_ESM.tif]

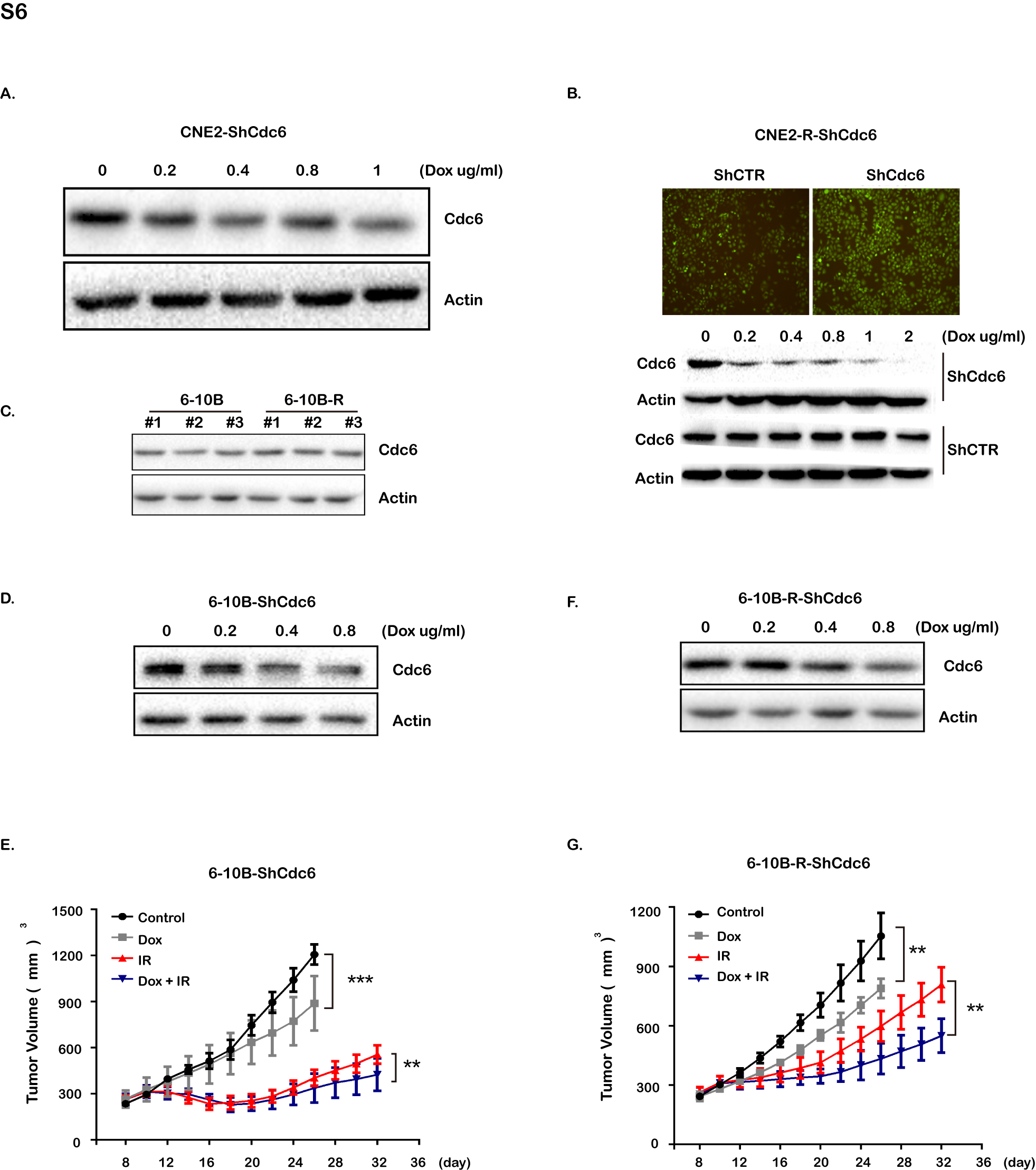

Supplement: Supplementary file 7 — Supplementary Figure 6 [file 41388_2018_460_MOESM7_ESM.tif]
